# Molecular Dissection of a Conserved Cluster of miRNAs Identifies Critical Structural Determinants That Mediate Differential Processing

**DOI:** 10.3389/fcell.2022.909212

**Published:** 2022-06-17

**Authors:** Manish Pandey, Arthur Luhur, Nicholas S. Sokol, Geetanjali Chawla

**Affiliations:** ^1^ RNA Biology Laboratory, Regional Centre for Biotechnology, Faridabad, India; ^2^ Department of Biology, Indiana University, Bloomington, IN, United States

**Keywords:** miRNA, terminal loop, stem-base, polycistronic, Drosha, Dicer

## Abstract

Differential processing is a hallmark of clustered microRNAs (miRNAs) and the role of position and order of miRNAs in a cluster together with the contribution of stem-base and terminal loops has not been explored extensively within the context of a polycistronic transcript. To elucidate the structural attributes of a polycistronic transcript that contribute towards the differences in efficiencies of processing of the co-transcribed miRNAs, we constructed a series of chimeric variants of *Drosophila let-7-Complex* that encodes three evolutionary conserved and differentially expressed miRNAs (*miR-100*, *let-7* and *miR-125*) and examined the expression and biological activity of the encoded miRNAs. The kinetic effects of Drosha and Dicer processing on the chimeric precursors were examined by *in vitro* processing assays. Our results highlight the importance of stem-base and terminal loop sequences in differential expression of polycistronic miRNAs and provide evidence that processing of a particular miRNA in a polycistronic transcript is in part determined by the kinetics of processing of adjacent miRNAs in the same cluster. Overall, this analysis provides specific guidelines for achieving differential expression of a particular miRNA in a cluster by structurally induced changes in primary miRNA (pri-miRNA) sequences.

## Introduction

MicroRNAs (miRNAs) are a class of small non-coding RNAs that regulate gene expression by directing effector complexes to target mRNAs (Bartel, 2009). These regulatory RNAs play critical roles in several biological processes, including development, differentiation, and cell fate determination (Kloosterman and Plasterk, 2006; Schickel et al., 2008; Ivey and Srivastava, 2010). The precise spatiotemporal control of miRNA levels is largely determined by the mechanisms that regulate biogenesis. Many of the miRNA loci reside in clusters that are transcribed as capped and polyadenylated primary transcripts (Lee et al., 2004). The expression and activity of miRNAs is regulated by transcription factors and post translational modifications of biogenesis factors (He et al., 2005; He et al., 2007; Kim et al., 2010). In addition to the rate of transcription, the processing efficiency of a miRNA precursor by Drosha and Dicer determine the level of the processed miRNA (Ha and Kim, 2014). The first step in the processing of a primary transcript is catalyzed by a nuclear protein complex referred to as the Microprocessor (Lee et al., 2003). The microprocessor complex is composed of the RNase III type enzyme Drosha, the double-stranded RNA binding protein, Pasha or DGCR8 in mammals and other auxiliary factors (Denli et al., 2004; Gregory et al., 2004; Han et al., 2004; Landthaler et al., 2004; Han et al., 2006). The primary miRNAs are cleaved by Drosha into hairpin structures (50–70 nucleotides long) referred to as precursor miRNAs (pre miRNAs). The pre miRNAs are then exported to the cytoplasm via interaction with the Exportin 5 receptor (Bohnsack et al., 2004; Lund et al., 2004). In the cytoplasm, the pre miRNAs are cleaved by another RNase III type enzyme referred to as Dicer 1 to form a miRNA duplex. In *Drosophila* the Dicer 1 functions together with the double stranded RNA binding protein Loquacious (Grishok et al., 2001; Ketting et al., 2001; Lim et al., 2016). The miRNA duplex associates with Argonaute (AGO) proteins to form the RNA-induced silencing complex (RISC). The AGO protein selects one strand of the duplex (referred to as the guide strand) and discards the other strand (passenger strand). The RISC scans mRNAs for miRNA binding sites and initiates silencing (Kobayashi and Tomari, 2016). A subsequent study that re-evaluated the roles of Drosha, Dicer and Exportin 5 found detectable miRNAs in Dicer and Exportin 5 knockout cells implying that alternate regulatory mechanisms exist to ensure miRNA biogenesis (Kim et al., 2016).

The processing efficiency of a pri-miRNA is determined by structural characteristics and sequence of the primary transcripts (Auyeung et al., 2013; Fang and Bartel, 2015; Adams, 2017; Roden et al., 2017). RNA binding proteins add another layer of regulation by recognizing specific sequences in the precursors and influence processing in different contexts (Heo et al., 2008; Choudhury and Michlewski, 2012; Treiber et al., 2019). In this study we have dissected the role of *cis* sequences in expression of a co-transcribed and evolutionary conserved cluster of miRNAs encoded by the *let-7-Complex* (*let-7-C*) in *Drosophila melanogaster*. The *let-7-C* locus encodes three evolutionary conserved miRNAs, miR-100, let-7 and miR-125 (Hertel et al., 2012; Sokol, 2012). We generated a series of chimeric UAS *let-7-C* cDNA constructs by swapping the position, stem base (B) and terminal loops (TL) of the pri-miR-100, pri-let-7 and pri-miR-125 transcripts. The expression of the three processed miRNAs was examined in transgenic flies and in a *Drosophila melanogaster* embryonic cell line Kc167. *In vitro* processing assays were performed with labeled chimeric transcripts to evaluate the kinetics of Drosha and Dicer processing. Furthermore, the functional activity of the chimeric constructs was assessed by miRNA sensor assays. Our results have identified critical structural determinants that are responsible for the differential expression of the *let-7-C* miRNAs.

## Materials and Methods

### Fly Strains and Husbandry

All fly strains were cultured on standard cornmeal medium at 25°C under 12 h light and 12 h dark cycles. Strains used were *let-7-*C^*GKI*^ and *let-7-C* ^*KO2*^(Sokol et al., 2008), and Bloomington Drosophila stock center, BDSC 3703 and BDSC 24871. The *let-7-C* ^*GKI*^ mutation contains a 991-base-pair deletion that removes the *miR-100*, *let-7*, and *miR-125*. Additionally, the *let-7-C* ^*GKI*^ mutation (*let-7-C GAL4 Knock-In*) contains the GAL-4 and *white* coding sequence driven by the *let-7-C* promoter (Sokol et al., 2008). The *let-7-C* ^*KO2*^ is identical except that the endogenous *let-7-C* locus was replaced with white rather than white and *gal4* (Wu et al., 2012). Flies of indicated genotypes were obtained by setting up standard genetic crosses. For genetic scheme refer to Supplementary Figure S1. Flies that were analyzed were trans-heterozygous for two different *let-7-C* null alleles (*let-7-C* ^*GKI*^ */let-7-C* ^*KO2*^), ensuring that phenotypes were not due to recessive mutations on either *let-7-C* mutant chromosome. In addition, third chromosomes that contained differing UAS transgenes were derived in parallel from the same population of flies. Finally, all flies had a common X-chromosome, derived from an isogenized stock. The UAS *let-7-C* cDNA transgenes were inserted into the VK00033 landing site (BDSC 24871). The genotypes of all the strains used in the study are indicated in the figure legends.

### Plasmids and Transgenes

#### Tagged Protein Plasmids

Plasmids encoding N-terminal Flag tagged version of Dicer were generated by recombining pENTR-Dicer (kind gift from Mikiko C. Siomi) with pTFW gateway plasmid (T. Murphy; obtained from DGRC) and the pAFW gateway plasmid (T. Murphy; obtained from DGRC) using the LR Clonase enzyme (ThermoFisher Scientific), respectively. The generation of flag-tagged Drosha and Pasha have been described in our previous study (Luhur et al., 2014).

#### UAS Transgenes

Pri-miR-100, pri-let-7 and pri-miR-125 hairpin wild type and chimeric constructs were generated by designing forward and reverse oligos encoding the precursor miRNA sequences as well as ∼50 nucleotides of conserved flanking sequences (See Supplementary Table S2 for oligo sequences). Oligo pairs with either Nhe1, Xba I or Avr II overhangs were annealed and cloned into the *XbaI* site of pUASTattB (a gift from Konrad Basler). The pri-let-7-C cDNA clone was generated by reverse transcription with total RNA extracted from DmBG3-c2 cells mixed with RNA from 24 h 20E treated Kc167 cells as described in our previous study (Chawla and Sokol, 2014). The reverse transcription was done with random hexamers and Splicing by Overlap extension polymerase chain reaction (SOE-PCR) with two sets of oligos (Horton et al., 1990). The PCR product was cloned into TOPO vector and then the *BamHI*-*XbaI* fragment was cloned into *BglII*-*XbaI* sites of pUASTattB. All PCRs were done with Pfu polymerase. The let-7-C cDNA chimeric constructs were generated by deleting the wild type pri-miRNAs and introducing AvrII, SpeI or XbaI restriction sites in the pri-miR-100, pri-let-7 and pri-mir-125 deletion sites. SOE-PCR was used to generate the cDNA construct with the deletion of the pri-miRNAs and insertion of restriction sites. The PCR product was cloned into the *XhoI-KpnI* sites of pLITMUS 28i vector (New England Biolabs). All chimeric hairpins were generated by annealing oligos with NheI overhangs. The annealed oligos were cloned into the AvrII, SpeI or XbaI sites. After ligating the hairpins, the cDNA was sub-cloned into pUASTattB.

### Luciferase Sensor Assays

**FIGURE 1:**
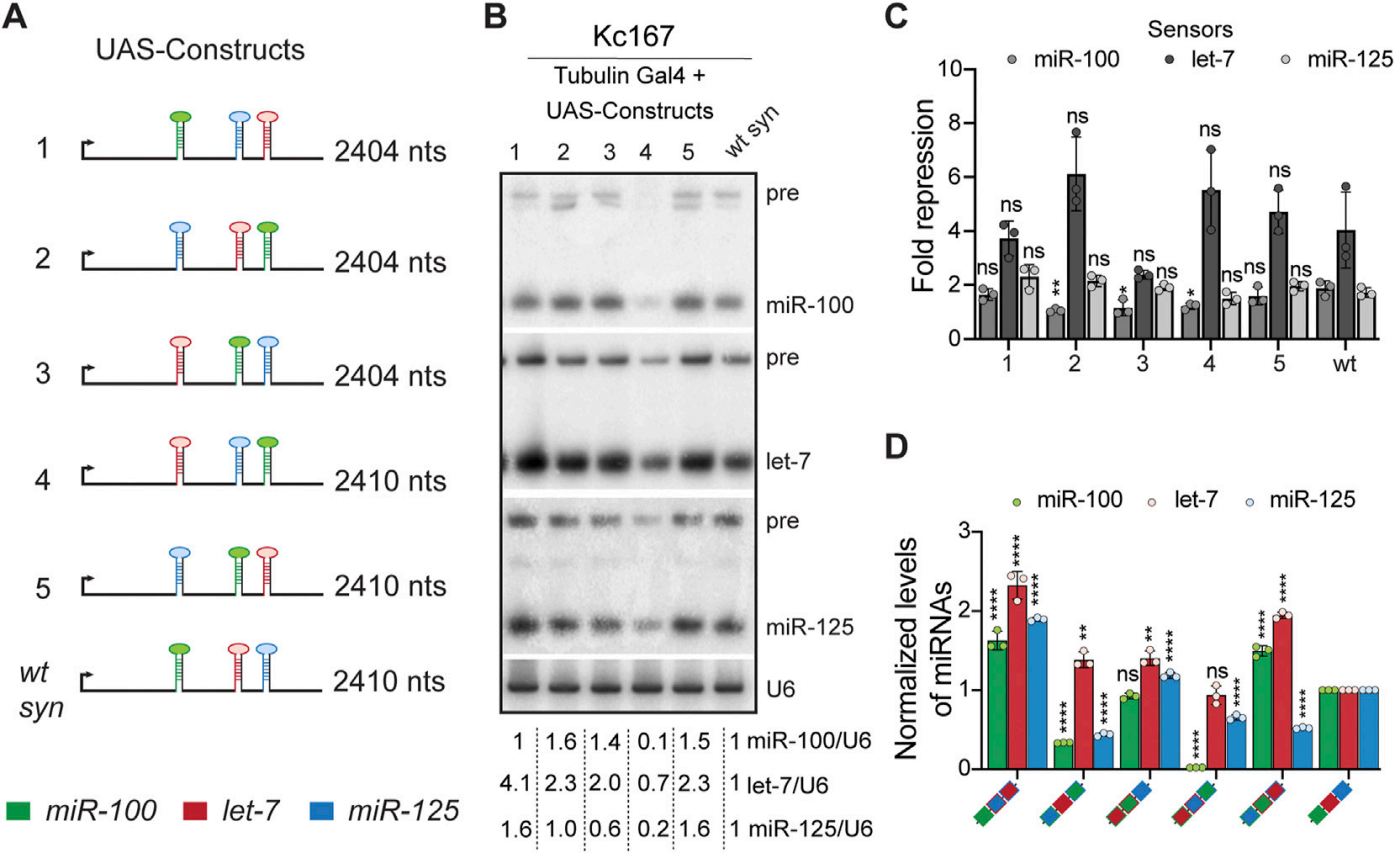
Positional context determines the processing of *let-7-Complex* (*let-7-C*) miRNAs. **(A)** Schematic representation of the *UAS let-7-C* constructs described in the figure panels **(B–D)**. **(B)** Small RNA northern blot analysis of Kc167 cells transfected with Tubulin Gal4 and *UAS let-7-C* expression plasmids indicated in **(A)**. Total RNA was extracted from Kc167 cells 72 h post-transfection. 10 μg of total RNA was resolved on three 15% urea-polyacrylamide gels and transferred onto hybridization membranes and probed for *miR-100*, *let-7* and *miR-125*. *U6snRNA* was used as a control for normalization and normalized levels of processed miRNAs are indicated below the gel. **(C)** Fold repression of *miR-100* (medium grey bar), *let-7* (dark grey bar) and *miR-125* (light grey bar) luciferase sensors in Kc167 cells transfected with *UAS let-7-C* cDNA constructs in **(A)**. Assays were performed in triplicates. Data are represented as mean ± SD, *n* = 3. *p*-value calculated by ordinary one-way ANOVA for miR-100 sensor is 3.30E-07; for let-7 sensor is 1.25E-09; and for miR-125 sensor is 1.91E-03. Adjusted *p*-values after applying Bonferroni’s correction are represented in the figure panel and we used an *α* level of 0.05 to assess statistical significance. **(D)** Expression of *miR-100* (green bar), *let-7* (red bar), and *miR-125* (blue bar) in transgenic lines expressing *UAS let-7-C* cDNA under the control of *let-7-C* Gal4 knock-in driver (*G4KI*) as determined by Taqman miRNA assays. The expression plasmids are variants of the let-7-C cDNAs where the position of miR-100, let-7 and miR-125 are interchanged and the changes are indicated in the color-coded legend. Total RNA was extracted from adult flies that were in a trans heterozygote *let-7-C* null mutant of the following genotype: *let-7-C* ^*GKI*^/*let-7-C* ^*KO2*^; *UAS transgene/+*. Assays were performed in triplicates. Data are represented as mean ± SD, *n* = 3. *p*-value calculated by ordinary one-way ANOVA for miR-100 is 1.06E-12; for let-7 is 7.19E-09; and for miR-125 is <1.00E-15. Adjusted *p*-values after applying Bonferroni’s correction are represented in the figure panel and we used an *α* level of 0.05 to assess statistical significance. 2S rRNA was used as a control for normalization. Genotype of strains used: (1D) *w* ^*1118*^ *; let-7-C* ^*GKI*^ */let-7-C* ^*KO2*^ *, P{neoFRT}40A; {w+, UAS let-7-C* ^*miR-100, miR-125, let-7*^ *} VK00033/+; w* ^*1118*^ *; let-7-C* ^*GKI*^ */let-7-C* ^*KO2*^ *, P{neoFRT}40A; {w+, UAS let-7-C* ^*miR-125, let-7, miR-100*^ *} VK00033/+; w* ^*1118*^ *; let-7-C* ^*GKI*^ */let-7-C* ^*KO2*^ *, P{neoFRT}40A; {w+, UAS let-7-C* ^*let-7, miR-100, miR-125*^ *} VK00033/+; w* ^*1118*^ *; let-7-C* ^*GKI*^ */let-7-C* ^*KO2*^ *, P{neoFRT}40A; {w+, UAS let-7-C* ^*let-7, miR-125, miR-100*^ *} VK00033/+; w* ^*1118*^ *; let-7-C* ^*GKI*^ */let-7-C* ^*KO2*^ *, P{neoFRT}40A; {w+, UAS let-7-C* ^*miR-125, miR-100, let-7*^ *} VK00033/+; w* ^*1118*^ *; let-7-C* ^*GKI*^ */let-7-C* ^*KO2*^ *, P{neoFRT}40A; {w+, UAS let-7-C* ^*miR-100, let-7, miR-125*^ *} VK00033/+*.

**FIGURE 2:**
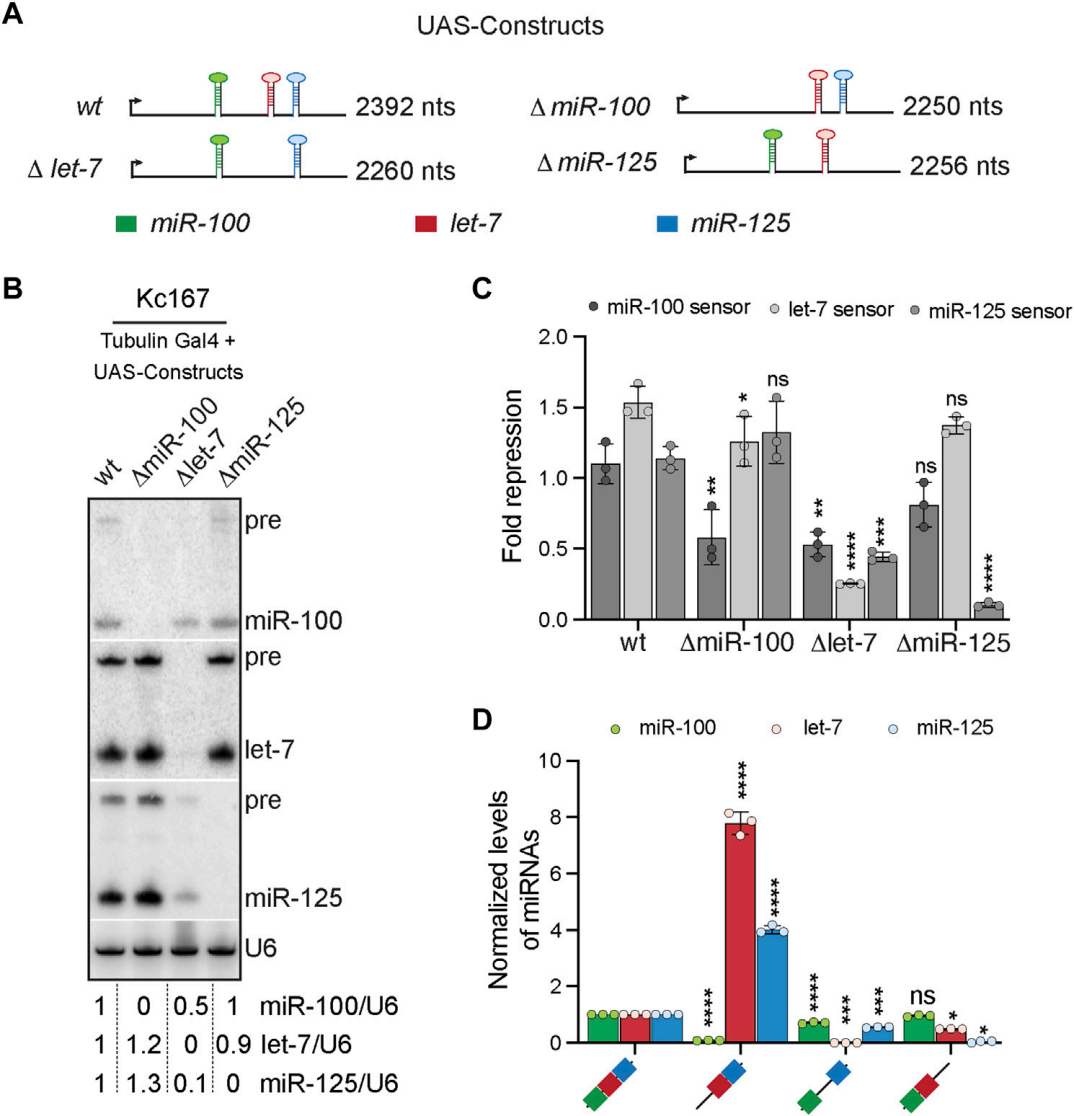
Cross-regulation of *let-7-Complex* (*let-7-C*) miRNAs. **(A)** Schematic of the *UAS let-7-C* constructs for determining the effect of adjacent hairpins in processing of *let-7-C* miRNAs. **(B)** Small RNA northern blot analysis of Kc167 cells transfected with Tubulin Gal4 and *UAS let-7-C* expression plasmids indicated in panel **(A)** and probed for *miR-100*, *let-7* and *miR-125*. *U6snRNA* was used as a control for normalization and normalized levels of each of the miRNAs are indicated below the gel. **(C)** Fold repression of *miR-100* (dark grey bar), *let-7* (light grey bar) and *miR-125* (medium grey bar) luciferase sensors in Kc167 cells transfected with *UAS let-7-C* cDNA constructs in panel **(A)**. Data are represented as mean ± SD, *n* = 3. *p*-value calculated by ordinary one-way ANOVA for miR-100 sensor is 5.9E-03; for let-7 sensor is 1.97E-06; and for miR-125 sensor is 4.31E-06. Adjusted *p*-values after applying Bonferroni’s correction are represented in the figure panel and we used an *α* level of 0.05 to assess statistical significance. **(D)** Expression of *miR-100* (green bar), *let-7* (red bar), and *miR-125* (blue bar) in transgenic lines expressing *UAS let-7-C* cDNA under the control of *let-7-C* Gal4 knock-in driver (*G4KI*) as determined by Taqman miRNA assays. The expression plasmids are variants of the *let-7-C* cDNAs where the miR-100, let-7 or miR-125 stem loops were deleted. Total RNA was extracted from adult flies that were in a trans heterozygote *let-7-C* null mutant of the following genotype: *let-7-C* ^*GKI*^/*let-7-C* ^*KO2*^ *; UAS transgene/+*. 2S rRNA was used as a control for normalization. Assays were performed in triplicates. Data are represented as mean ± SD, *n* = 3. *p*-value calculated by ordinary one-way ANOVA for miR-100 is 9.10 E-11; for let-7 is 1.27E-10; and for miR-125 is 1.28E-11. Adjusted *p*-values after applying Bonferroni’s correction are represented in the figure panel and we used an *α* level of 0.05 to assess statistical significance. Genotype of strains used: (2D) *w* ^*1118*^ *; let-7-C* ^*GKI*^ */let-7-C* ^*KO2*^ *, P{neoFRT}40A; {w+, UAS let-7-C* ^*miR-100, let-7, miR-125*^ *} VK00033/+; w* ^*1118*^ *; let-7-C* ^*GKI*^ */let-7-C* ^*KO2*^ *, P{neoFRT}40A; {w+, UAS let-7-C* ^*ΔmiR-100*^ *} VK00033/+; w* ^*1118*^ *; let-7-C* ^*GKI*^ */let-7-C* ^*KO2*^ *, P{neoFRT}40A; {w+, UAS let-7-C* ^*Δlet-7*^ *} VK00033/+; w* ^*1118*^ *; let-7-C* ^*GKI*^ */let-7-C* ^*KO2*^ *, P{neoFRT}40A; {w+, UAS let-7-C* ^*ΔmiR-125*^ *} VK00033/+*.

**FIGURE 3:**
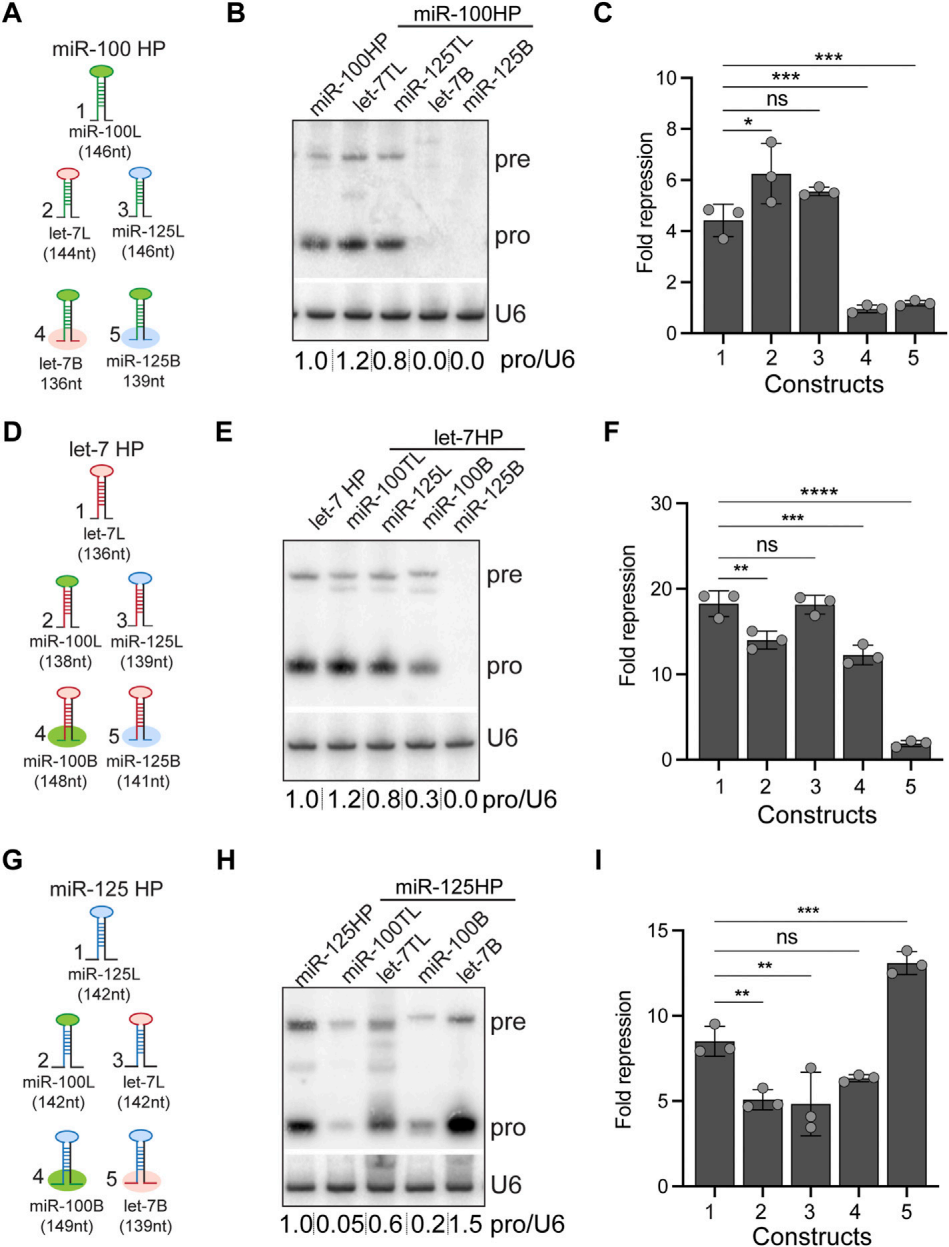
Expression and activity of *let-7-Complex* pri-miRNA monocistronic chimeras. **(A)** Schematic representation of UAS constructs used for experiments indicated in panels **(B,C)**. **(B)** Small RNA northern blot of total RNA extracted from Kc167 cells transfected with Tubulin Gal4 and one of the following constructs: UAS pri-miR-100, UAS pri-miR-100H let-7 terminal loop (TL) chimera, UAS pri-mir-100H miR-125TL chimera, UAS pri-miR-100H let-7B, or UAS pri-miR-100HmiR-125B. The Northern blot was probed for *miR-100* and *U6 snRNA* was used as normalization control. **(C)** Luciferase sensor assay analysis to determine the functional activity of the UAS pri-miR-100 chimeric constructs. Fold repression of *miR-100* sensor in Kc167 cells transfected with *UAS* constructs represented in panel **(A)**. Data are represented as mean ± SD, *n* = 3. *p*-value calculated by ordinary one-way ANOVA for miR-100 sensor is 1.51E-06. Adjusted *p*-values after applying Bonferroni’s correction are represented in the figure panel and we used an *α* level of 0.05 to assess statistical significance. **(D)** Schematic representation of UAS constructs used for experiments indicated in panels **(E,F)**. **(E)** RNA northern blot of total RNA extracted from Kc167 cells transfected with Tubulin Gal4 and one of the following constructs: UAS pri-let-7, UAS pri-let-7HmiR-100TL chimera, UAS pri-let-7HmiR-125TL, UAS pri-let-7HmiR-100B, or UAS pri-let-7HmiR-125B. The Northern blot was probed for *let-7* and *U6 snRNA* was used as normalization control. **(F)** Luciferase sensor assay analysis to determine the functional activity of the UAS pri-let-7 chimeric constructs. Fold repression of *let-7* sensor in Kc167 cells transfected with Tubulin Gal4 and *UAS* constructs represented in panel **(G)**. Data are represented as mean ± SD, *n* = 3. *p*-value calculated by ordinary one-way ANOVA for *let-7* sensor is 3.00E-08. Adjusted *p*-values after applying Bonferroni’s correction are represented in the figure panel and we used an *α* level of 0.05 to assess statistical significance. **(G)** Schematic representation of UAS constructs used for experiments indicated in panels **(H,I)**. **(H)** RNA northern blot of total RNA extracted from Kc167 cells transfected with Tubulin Gal4 and one of the following constructs: UAS pri-miR-125, UAS pri-miR-125 miR-100TL chimera, UAS pri-miR-125 let-7TL, UAS pri-miR-125HmiR-100B, or UAS pri-miR-125Hlet-7B. The Northern blot was probed for *miR-125* and *U6 snRNA* was used as a control for normalization. **(I)** Luciferase sensor assay analysis to determine the functional activity of the UAS pri-miR-125 chimeric constructs. Fold repression of *miR-125* sensor in Kc167 cells transfected with Tubulin Gal4 and *UAS* constructs represented in panel **(G)**. Data are represented as mean ± SD, *n* = 3. *p*-value calculated by ordinary one-way ANOVA for *miR-125* sensor is 8.03E-06. Adjusted *p*-values after applying Bonferroni’s correction are represented in the figure panel and we used an *α* level of 0.05 to assess statistical significance.

*Drosophila* Kc167 cells were cultured in CCM3 at 23°C. For sensor assays, psiCHECK plasmids (50 ng/well) bearing six perfect sites for either miR-100, let-7 or miR-125 downstream of a *Renilla* luciferase gene were cotransfected with a Tubulin-GAL4 plasmid (50 ng/well) or Ubiquitin-GAL4 plasmid as well as plasmids encoding either unmodified or edited versions of UAS pri-let-7-C (50 ng/well) in Kc167 cells in 48-well plates. After 72 h, luciferase activity was measured with the Dual-Glo luciferase Assay system (Promega). Fold repression was calculated by dividing the ratio of *Renilla* luciferase and firefly Luciferase in cells transfected with an empty pUAST attB plasmid with the ratio of *Renilla* luciferase and firefly luciferase in cells transfected with pUAST attB plasmid containing *let-7-C* cDNAs. The luciferase reporter assay in Figure 1C and Figure 2C was performed using 25 μl cell lysate in a 96-well format and was quantitated using a GLOMAX 96 microplate luminometer. For Figures 3C,F,I, assays were performed with 50 μl cell lysate in a Turner Model TD-20/20 luminometer.

### Purification of Flag Tagged Proteins

Kc167 cells were cotransfected with expression plasmids for Flag-Drosha and Flag-Pasha or Flag-Dicer with effectene transfection reagent (Qiagen). Cells were plated onto 10-cm petridishes at a density of 1 × 10^6^ cells/ml, transfected with 6 μg of Flag Drosha together with 4 μg of Flag Pasha plasmid DNA or 10 μg of Flag Dicer plasmid along with 50 μl of Effectene per petridish. The cells were harvested after 72 h. Complexes were purified form cell lysates with anti-Flag M2 affinity gel (Sigma Aldrich) according to manufacturer’s instructions. The complex/protein was eluted with 400 μg/ml of 3X Flag peptide (Sigma Aldrich). The eluate was concentrated with Microcon concentrator column (Millipore).

### *In Vitro* Drosha and Dicer Processing Assays

DNA template for transcription was generated by PCR with the T7 and 2162 oligo pairs for pri-let-7 transcripts, 2175/2186 for pri-miR-100 transcripts, and T7/2164 for pri-miR-125 transcripts (Refer to Supplementary Table S2 oligonucleotide sequences). Primary transcripts were transcribed and labelled with ^32^UTP (Perkin Elmer) using the T7 Megashortscript Kit (Invitrogen). The transcript was purified by running the DNAse treated reaction on a 4% denaturing PAGE gel and the gel piece corresponding to the labeled transcript was excised from the gel and eluted in an Eppendorf Thermomixer (400 rpm) at 37°C in a buffer containing 0.3 M Sodium acetate, 0.2% Sodium dodecyl sulphate, and 1 mM EDTA. The supernatant was precipitated in ethanol. The precipitated RNA was refolded by heating at 95°C for 2 min followed by 37°C for 1 h. A typical 25 μl reaction contained 15 μl of the Flag-Drosha-Pasha beads immunoprecipitate, 6.4 mM MgCl_2_, 1 U/μl of Ribonuclease Inhibitor (Invitrogen), and the refolded labeled transcripts (0.5 × 10^5^ cpm). The reaction mixture was incubated at 26°C for 30–90 min, and RNA was extracted by phenol followed by ethanol precipitation and analyzed on a 10% denaturing polyacrylamide gel.

*In vitro* dicing assays were performed as described in our previous study (Chawla et al., 2016). Briefly, 25 nM purified Flag Tagged Dicer protein was combined with 1 nM 5′-radiolabeled substrate RNAs, 5% (v/v) Glycerol, 1 mM DTT, 0.1-unit RNAse Inhibitor (ThermoFisher Scientific) and incubated for 10–60 min. After completion of the incubation time, the reaction products were resolved by electrophoresis on a 10% denaturing PAGE gel, followed by drying and detection by Typhoon scanner and quantified by Image-Quant software.

### Quantitative Real Time PCR

Total RNA was extracted with Trizol and treated with DNAse I. The purified RNA was used in reverse transcription using Superscript III (Thermo Fisher Scientific). The first-strand cDNA was used as a template for qRT-PCR in a volume of 10–15 μl containing oligos and Taqman Universal PCR master mix. For mature miRNAs, expression levels were measured by qRT-PCR analysis with TaqMan miRNA assays containing specific oligos for mature miR-100, let-7 and mIR-125 (Thermo Fisher Scientific) using a StepOnePlus or QuantStudio 6 Real time PCR machine (Thermo Fisher Scientific). A standard curve was run in each PCR. Individual values were normalized to 2S rRNA levels for Taqman miRNA assays. All reactions were done three times, and relative expression of RNAs was calculated using the Pfaffl method (Pfaffl, 2001).

### Northern Blot Analysis

Northern blot analysis was performed as described previously (Chawla and Sokol, 2012). 10 μg of total RNA was resolved on a 15% urea-polyacrylamide gel and transferred onto Genescreen plus hybridization membrane (Perkin Elmer). StarFire oligos (IDT) were end-labeled and used as probes for northern analysis. Northern blots were exposed to a PhosphorImager screen and visualized by ImageQuant 5.1 software (Molecular Dynamics). For Northern blot analysis in Kc167 cells, each of the pUAST attB hairpin constructs (600 ng/plate) was co-transfected with Tubulin-GAL4 (600 ng/plate) for 68–72 h in 60 mm plates.

### Statistical Analyses

Quantified data are expressed as the mean ± SD values. An ordinary one-way ANOVA was used to analyze data from RT-PCR and luciferase sensor assays where multiple comparisons were made. GraphPad Prism and Microsoft Excel was used for statistical analysis. All RT-PCR analysis was performed with three independent biological replicates and individual data points were plotted in all graphs. Adjusted *p*-values for all comparisons were computed by applying Bonferroni’s correction and noted in the figure panels. Statistical significance was set at *p* < 0.05. For all figures, ∗*p* < 0.05, ∗∗*p* < 0.01, ∗∗∗*p* < 0.001, and ∗∗∗∗*p* < 0.0001.

## Results

### Position and Order of *let-7-Complex* miRNAs Are Critical Determinants for Processing

The genomic clustering and the relative ordering of miR-100, let-7, and miR-125 are phylogenetically conserved (Sempere et al., 2003; Wulczyn et al., 2007; Roush and Slack, 2008; Sokol et al., 2008; Hertel et al., 2012; Sokol, 2012; Mohammed et al., 2014). Due to this clustering, these miRNAs are co-transcribed and co-regulated in *Drosophila melanogaster* (Chawla and Sokol, 2012). Another key feature of *let-7-C* primary transcript is the relative conservation of the order of the three miRNAs. The pri let-7-C transcript is an ∼ 2.2 kb transcript that harbors pre-miR-100 (61 nt), pre-let-7 (61 nt) and pre-miR-125 (60 nt) (Supplementary Table S1). For the ease of readability, we have used the terms pri-miR-100 (146 nt), pri-let-7 (138 nt) and pri-miR-125 (142 nt) to describe precursor sequences flanked by conserved sequences (For sequences refer to Supplementary Table S1). To examine the importance of the position of a particular primary miRNA in the processing of the three miRNAs in the cluster, we generated *UAS-let-7-C* cDNA constructs in which the position of either two (constructs 1, 2 and 3) or all three (constructs 4 and 5) primary miRNAs (pri-miRNAs) in *let-7-C* cDNA were interchanged (Figure 1A). The expression of these constructs was examined in the Kc167 cell line that does not express *let-7-C* endogenously. Since Kc167 cells do not express let-7-C miRNAs in absence of Ecdysone, we cotransfected UAS chimeric constructs with Tubulin Gal4 to uncouple transcriptional control from post-transcriptional control of the cluster. As control, we utilized a wild type UAS *let-7-C* construct in which the three pri-miRNAs were re-inserted in their native positions after introducing restriction enzyme sites (as described in *Materials and Methods*) used for introducing the chimeric hairpins. This construct was refered to as wild type synthetic construct (wt syn) (Figure 1A). The expression of these constructs was also examined in transgenic flies containing the *UAS let-7-C* cDNA constructs under the control of *let-7-C* GAL 4 (*let-7-C* ^*GKI*^) driver in a *let-7-C* mutant background. The *let-7-C* ^*GKI*^ mutation contains a 991-base-pair deletion that removes the *miR-100*, *let-7*, and *miR-125*. Additionally, the *let-7-C* ^*GKI*^ mutation (*let-7-C GAL4 Knock-In*) contains the GAL-4 and *white* coding sequence driven by the *let-7-C* promoter (Sokol et al., 2008). Using phiC31-mediated integration, these transgenes were inserted into identical chromosomal locations and crossed into a trans-heterozygous *let-7-C* null background to yield the experimental strains (For genetic scheme refer to Supplementary Figure S1). The levels of each of these processed miRNAs was examined by Northern blots and Real-time Quantitative Reverse transcription PCR (qRT-PCR), respectively. Quantitation of the bands in the Northern blot indicated that miR-100 was reduced to 10%, let-7 was reduced to 70% and miR-125 was reduced to 20% of the levels in the wild type construct when construct 4 was transfected (let-7-miR-125-miR-100). Expression of let-7 and miR-125 was increased when constructs 1 (miR-100-miR-125-let-7) and 5 (miR-125-miR-100-let-7) were transfected (Figures 1A,B). An increase in miR-100 and let-7 levels was observed upon transfection of constructs 2 and 3 (Figures 1A,B). To determine whether the position of a pri-miRNA in *let-7-C* resulted in changes in the degree of repression by the mature miRNAs, luciferase sensor assays were performed with the different UAS *let-7-C* cDNAs (Figure 1C). The degree of repression of miR-100 sensor was decreased for constructs 2, 3 and 4. This indicates that the amongst the three *let-7-C* miRNAs, the degree of repression of miR-100 was determined by the position of the pri-miR-100 in the cluster. The degree of repression of let-7 and miR-125 sensors was not significantly altered in any of the configurations tested. To assess whether the position of the miRNA hairpins influenced expression of the miRNAs *in vivo*, the expression of the three miRNAs was examined in transgenic *Drosophila* lines expressing a single copy of the UAS transgene under the control of the *let-7-C* promoter (Figure 1D). Transgenic analysis revealed that the level of processed miR-100, and miR-125 was significantly reduced in transgenic lines (2% and 62% relative to the wild type transgene) expressing construct 4 (let-7-miR-125-miR-100) (Figure 1D). Expression of miR-100 in the transgenic lines was not significantly different from wild type control (construct 3) or higher than wild type (construct 1 and construct 5) when it was placed in either position 1 or 2 of pri-let-7-C (Figure 1D). However, placing pri-let-7 in position 3 enhanced miR-100 (Construct 1: 1.6-fold increase; Construct 5: 1.4-fold increase) and let-7 (construct 1: 2.3-fold increase and construct 5: 1.9-fold increase). The transgenic line in which pri-miR-125 and pri-let-7 were swapped resulted in an increased expression of all three miRNAs (Construct 1) (Figure 1D).

In summary, this analysis revealed that changing the order of miRNAs to miR-100-miR-125-let-7 resulted in more efficient processing of all the three miRNAs in the *let-7-C* cluster. Swapping of pri-miR-100 and pri-let-7 (let-7-miR-100-miR-125) also resulted in a favorable configuration where let-7 and miR-125 levels were significantly increased relative to the wildtype and miR-100 expression was not altered. *In vitro* sensor assays did not reveal an increased degree of functional activity in configurations where let-7 and miR-125 levels were increased. This could be due to saturation of the sensors; however, we were not able to observe an increase in fold repression even upon reduction in the transfected constructs. *In vivo* expression analysis of the chimeras revealed configurations where the expression level of all three miRNAs was higher than the control (Construct 1). However, the increase observed was in the range of 1.5-2-fold. Future identification of mRNA targets that are regulated by all three *let-7-C* miRNAs in combination or exclusively will aid in further understanding the role of positional context *in vivo*.

### Absence of let-7 Reduces Processing of miR-100 and miR-125

To address whether presence of a miRNA influences the expression, processing and/or function of the adjacent miRNAs in the *let-7-C* cluster we generated UAS *let-7-C* cDNA constructs in which each of the pri-miRNA sequence was deleted (Figure 2A). Northern blot analysis of *UAS let-7-C* cDNA constructs and luciferase sensor assays were performed in Kc167 cells to determine the levels of the functional mature miRNAs (Figures 2B,C). Small RNA northern blot analysis revealed that absence of miR-100 stem loop resulted in a slight increase (1.2–1.3-fold) in expression of both let-7 and miR-125 (Figure 2B). However, this increase did not lead to an increased repression of the luciferase let-7 or miR-125 sensor *in vitro* (Figure 2C). In contrast, deletion of the pri-let-7 resulted in a 50% reduction in miR-100 levels and a 90% reduction in miR-125 levels and a concomitant 50% decrease in the repression of the miR-100 sensor and a 69% decrease in the repression of miR-125 sensor (Figure 2C). Deletion of the miR-125 stem loop led to a 10% reduction in let-7 levels with no effect on the repression of let-7 sensor in Kc167 cells. Expression of the three miRNAs in transgenic lines expressing a single copy of the transgene under the control of the *G4KI* was quantitated by RT-PCR to examine the effect of deletion of each of the three pri-miRNAs (Figure 2D). Deletion of pri-miR-100 resulted in 770% increase in the levels of processed let-7 and a 400% increase in processed miR-125 levels, respectively. Consistent with the *in vitro* analysis in Kc167 cell line, deletion of pri let-7 resulted in 30% decrease in mature miR-100 and a 45% decrease in mature miR-125 levels (Figure 2D). Taken together, this analysis revealed that the presence of pri-let-7 determines processing and subsequent expression of miR-100 and miR-125. Our previous analysis had shown that deletion of pri-let-7 significantly altered the levels of miR-100 and miR-125 (Chawla et al., 2016). Our analysis with UAS let-7-C cDNA variants has uncovered a differential role of each of the stem loops in regulation of the adjacent miRNAs. We show that the absence of pri-miR-100 (ΔmiR-100), enhances expression of processed let-7 and miR-125, the deletion of pri-let-7 (Δlet-7) decreases expression of miR-100 and miR-125 and deletion of pri-miR-125 (ΔmiR-125) reduces expression of let-7 (Figures 2B,D). Thus, highlighting the contribution of each of the stem loops in the conserved polycistronic locus.

### Processing of Pri-miR-125 and Not Pri-miR-100 Can Be Enhanced by Substitution With the Pri-let-7 Stem-Base

Most canonical pri-miRNAs consist of four structural features: an upper stem region that harbors the miRNA duplex, a terminal loop, the miRNA duplex, and the lower stem and the flanking single stranded basal sequences (Han et al., 2006; Zeng and Cullen, 2006). To determine the relative contribution of the stem-bases and terminal loops of pri-miR-100, pri-let-7 and pri-miR-125 in differential expression of the three miRNAs, we generated chimeras of all three pri-miRNAs in *let-7-C* in which the stem-base sequence or the terminal loop of a particular pri-miRNA was swapped with that of the other two (Figures 3A,D,G). The length of the regions of the stem-base sequence to be swapped were based on the structures represented for these pri-miRNAs in miRbase website and sequence conservation between *Drosophila* species (Griffiths-Jones, 2004; Griffiths-Jones et al., 2006; Griffiths-Jones et al., 2008; Kozomara and Griffiths-Jones, 2011; Kozomara and Griffiths-Jones, 2014; Kozomara et al., 2019). We hypothesized that changes in the stem-base sequence would lead to changes in the secondary structure and hence processing of the primary miRNAs. The Mfold predicted structures for the wild type and chimeric miRNAs are represented in Supplementary Figures S2, S3 (Zuker and Jacobson, 1998; Waugh et al., 2002; Zuker, 2003). The Mfold analysis revealed that changes in the stembase and terminal loops altered pri-miRNA and pre-miRNA structures (Supplementary Figures S2, S3). Since these predictions do not consider the effects of RNA-binding auxiliary factors and biomolecules that likely occurs within cells, other assays were utilized to ascertain changes in processing of the pri-miRNAs. The expression and activity of the chimeric constructs was analyzed by transfecting Kc167 cells with the expression plasmids along with Tubulin Gal4. Total RNA was extracted from transfected cells and levels of pre-miRNA and processed miRNA were analyzed by small RNA northern analysis (Figures 3B,E,H). Substitution of pri-miR-100 stem-base with the stem-base of either pri-let-7 or pri-miR-125 diminished expression of miR-100 (Figure 3B). In contrast, terminal loop chimera in which miR-100 terminal loop was substituted with pri-let-7 terminal loop (pri-miR-100Hlet-7L) was expressed at 1.2-fold higher levels as compared to the pri-miR-100 hairpin and a significant increase in repression of the miR-100 sensor (Figure 3C). The substitution of pri-miR-100 terminal loop with pri-miR-125 terminal loop (pri-miR-100HmiR-125L) resulted in a 0.2-fold decrease in miR-100 expression with no significant change in fold repression of miR-100 sensor in Kc167 cells (Figure 3C). Substitution of pri-let-7 terminal loop with pri-miR-100 terminal loop (pri-let-7HmiR-100L) resulted in a 0.2-fold increase in let-7 levels by Northern blot analysis, but a 25% decrease in fold repression of the let-7 sensor (Figures 3E,F). This was likely due to the higher levels of expression of let-7 and the reduced sensitivity of the Northern. However, Taqman RTPCR in transgenic monocistronic line revealed a decrease in expression of pri-let-7HmiR-100B compared to the wild type pri-let-7. Swapping the terminal loop of pri-miR-125 with the terminal loop of pri-miR-100 (pri-miR-125HmiR-100L) or pri-let-7 (pri-miR-125Hlet-7L) resulted in a 99.05% and 40% decrease in the expression of mature miR-125 and a concomitant 40% and 44% decrease in the repression of the miR-125 sensor, respectively (Figures 3G–I). Substituting the stem-base of pri-miR-125 with that of pri-miR-100 resulted in an 80% decrease in the levels of mature miR-125 and a 25% decrease in repression of the miR-125 sensor (Figures 3H,I). Strikingly, substituting the stem-base of pri-miR-125 with the stem-base of pri-let-7 led to 150% increase in processed miR-125 and a 5-fold increase in repression of the miR-125 sensor (Figures 3H,I). The small RNA northern blot analysis also revealed differences in the band size of the pre-miR-125 bands in the stem-base chimeras pri-let-7HmiR-100B and pri-miR-125HmiR-100B (Figures 3E,H). Thus, suggesting that the monocistronic pri-miRNA is cleaved by Drosha at alternate sites that resulted in the generation of isomiRs that were not able to repress the canonical sensor efficiently.

**FIGURE 4:**
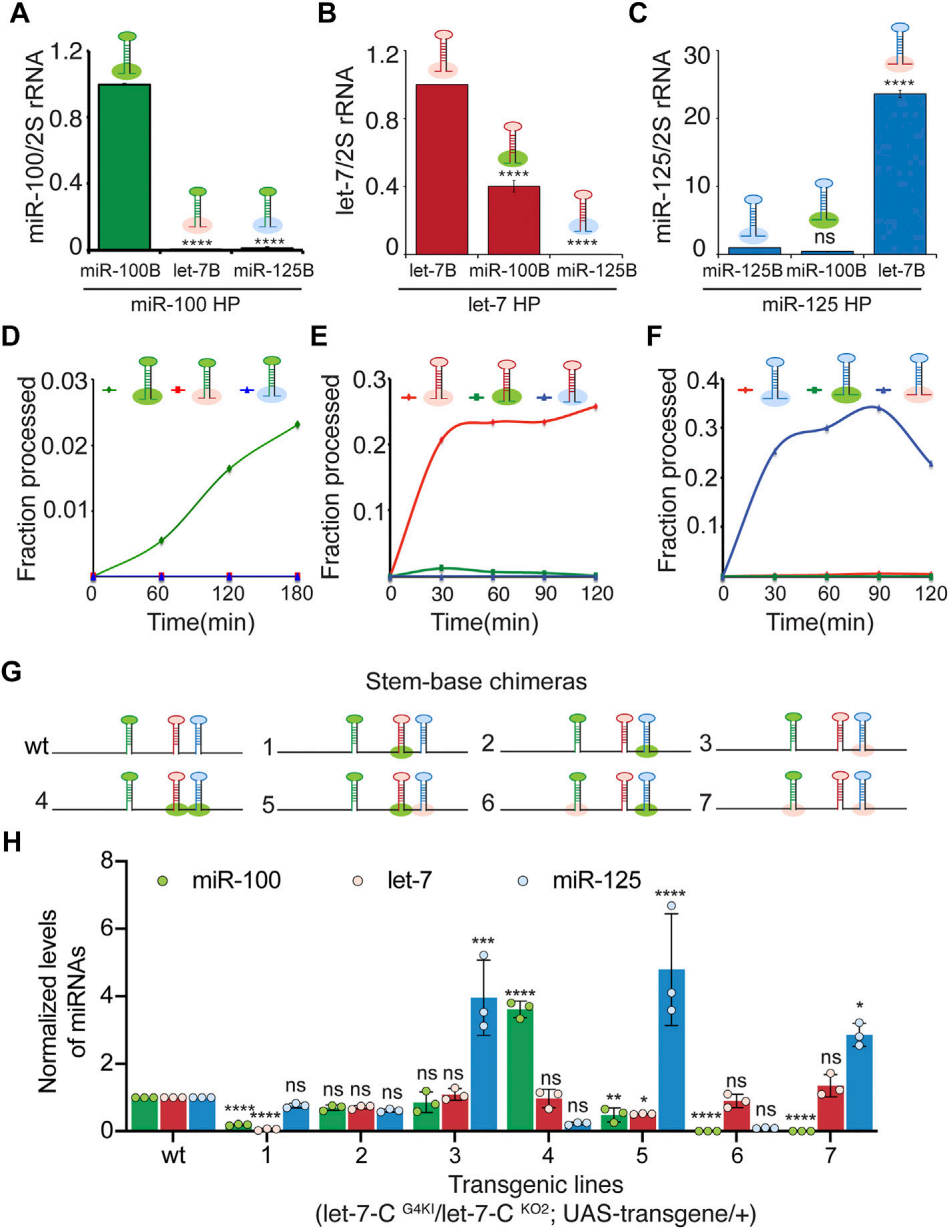
The basal stem sequences regulate the expression and function of the *let-7-C* miRNAs. **(A)** Expression of miR-100 in transgenic lines expressing *UAS pri*-*miR-100, UAS pri-miR-*100Hlet*-7B*, *and UAS pri-mir-100HmiR-125B* under the control of *let-7-C* Gal4 knock-in driver (*G4KI*) as determined by Taqman miRNA assays. Data are represented as mean ± SD, *n* = 3. *p*-value calculated by ordinary one-way ANOVA is 7.57E-13. **(B)** Expression of let-7 in transgenic lines expressing *UAS pri*-*let-7, UAS pri-let-7HmiR-100B, and UAS pri-let-7HmiR-125B* under the control of *let-7-C* Gal4 knock-in driver (*G4KI*) as determined by Taqman miRNA assays. Data are represented as mean ± SD, *n* = 3. *p*-value calculated by ordinary one-way ANOVA is 1.15E-08. **(C)** Expression of miR-125 in transgenic lines expressing *UAS pri-miR-125, UAS pri-miR-125HmiR-100B, and UAS pri-miR-125Hlet-7B* under the control of *let-7-C* Gal4 knock-in driver (*G4KI*) as determined by Taqman miRNA assays. Data are represented as mean ± SD, *n* = 3. *p*-value calculated by ordinary one-way ANOVA is 7.20E-10. **(D–F)** Line graph depicting kinetics of processing of wild type (green line) and chimeric pri-miR-100 (blue and red) **(D)**, wild type (red line) and chimeric pri-let-7 (green and blue) **(E)**, and wild type (red line) and chimeric pri-miR-125 (blue and green) **(F)**. **(D–F)** Chimeric and wild type transcripts were subjected to Drosha cleavage, and the fraction of processed pre-miRNA was calculated as the ratio of pre/pre+pri. **(G)** Schematic representation of UAS *let-7-C* chimeras that are described in panel **(H)**. **(H)** Expression of *miR-100* (green bar), *let-7* (red bar), and *miR-125* (blue bar) in transgenic lines expressing *UAS let-7-C* cDNA wild type and chimeras under the control of *let-7-C* Gal4 knock-in driver (*G4KI*) as determined by Taqman miRNA assays. Total RNA was extracted from adult flies that were in a trans heterozygote *let-7-C* null mutant of the following genotype: *let-7-C* ^*GKI*^/*let-7-C* ^*KO2*^; *UAS transgene/+*. 2S rRNA was used as a control for normalization. Data are represented as mean ± SD, *n* = 3. *p*-value calculated by ordinary one-way ANOVA for miR-100 is 1.22 E-13; for let-7 is 6.36E-06; and for miR-125 is 1.06E-06. Adjusted *p*-values after applying Bonferroni’s correction are represented in the figure panel and we used an *α* level of 0.05 to assess statistical significance. Genotype of strains used: *(4A)* *w* ^*1118*^ *; let-7-C* ^*GKI*^ */let-7-C* ^*KO2*^ *, P{neoFRT}40A; {w+, UAS miR-100} VK00033/+; w* ^*1118*^ *; let-7-C* ^*GKI*^ */let-7-C* ^*KO2*^ *, P{neoFRT}40A; {w+, UAS miR-100Hlet-7B} VK00033/+*; *w* ^*1118*^ *; let-7-C* ^*GKI*^ */let-7-C* ^*KO2*^ *, P{neoFRT}40A; {w+, UAS miR-100HmiR-125B}VK00033/+ ;(4B) w^1118^; let-7-C^GKI^ / let-7-C^KO2^, P{neoFRT}40A; {w+, UAS let-7} VK00033/+; w^1118^; let-7-C^GKI^ / let-7-C^KO2^, P{neoFRT}^40A^; {w+, UASlet-7HmiR-100B} VK00033/+; w^1118^; let-7-C^GKI^ / let-7-C^KO2^, P{neoFRT}40A; {w+, UAS let-7HmiR-125B}VK00033/+ ; (4C) w^1118^; let-7-C^GKI^ / let-7-C^KO2^, P{neoFRT}40A; {w+, UAS miR-125} VK00033/+; w^1118^; let-7-C^GKI^ / let-7-C^KO2^, P{neoFRT}^40A^; {w+, UAS miR-125HmiR-100B} VK00033/+; w^1118^; let-7-C^GKI^ / let-7-C^KO2^, P{neoFRT}^40A^; {w+, UAS miR-125Hlet-7B}VK00033/+;(4I)* *w* ^*1118*^ *; let-7-C* ^*GKI*^ */let-7-C* ^*KO2*^ *, P{neoFRT}40A; {w+, UAS let-7-C* ^*miR-100, let-7, miR-125*^ *} VK00033/+; w* ^*1118*^ *; let-7-C* ^*GKI*^ */let-7-C* ^*KO2*^ *, P{neoFRT}40A; {w+, UAS let-7-C* ^*miR-100, let-7HmiR-100B, miR-125*^} *VK00033/+*; *w* ^*1118*^; *let-7-C* ^*GKI*^ */let-7-C* ^*KO2*^, *P{neoFRT}40A; {w+, UAS let-7-C* ^*miR-100, let-7, miR-125HmiR-100B*^} VK00033/+; w^1118^; let-7-C^GKI^/let-7-C^KO2^, P{neoFRT}40A; {w+, UAS let-7-C ^miR-100, let-7, miR-125Hlet-7B^} VK00033/+; w^1118^; let-7-C^GKI^/let-7-C^KO2^, P{neoFRT}40A; {w+, UAS let-7-C ^miR-100, let-7HmiR-100B, miR-125HmiR-100B^} VK00033/+; w^1118^; let-7-C^GKI^/let-7-C^KO2^, P{neoFRT}40A; {w+, UAS let-7-C ^miR-100, let-7HmiR-100B, miR-125Hlet-7B^} VK00033/+; w^1118^; let-7-C^GKI^/let-7-C^KO2^, P{neoFRT}40A; {w+, UAS let-7-C ^miR-100let-7B, let-7, miR-125HmiR-100B^} VK00033/+; w^1118^; let-7-C^GKI^/let-7-C^KO2^, P{neoFRT}40A; {w+, UAS let-7-C ^miR-100let-7B, let-7, miR-125Hlet-7B^} VK00033/+.

To examine the expression of the stem-base chimeras *in vivo*, we utilized a more sensitive Taqman miRNA real time PCR assay to quantitate the expression of the processed *let-7-C* miRNAs in 3-day old adult flies (Figures 4A–C). Consistent with the Kc167 cell line data, substitution of pri-miR-100 stem base with stem base from pri-let-7 (0.08% compared to wild type) or pri-miR-125 (1% compared to wild type) resulted in a significant decrease in expression of miR-100 (Figure 4A). Replacing pri-let-7 stem base with pri-miR-100 stem base (40% relative to wild type) or pri-miR-125 stem base (0.004%) also led to a significant reduction in processed let-7 levels (Figure 4B). In contrast, substitution of pri-miR-125 with pri-let-7 stem base resulted in 23-fold (2300% increase relative to wild type pri-miR-125) increase in the expression of miR-125 levels (Figure 4C). However, substitution of pri-miR-125 stem base with pri-miR-100 stem base resulted in a reduction in expression of mature miR-125 (40% relative to wild type) (Figure 4C).

The cleavage of primary miRNAs by the microprocessor (Drosha-Pasha) complex is the initiating step of the canonical biogenesis pathway and results in the generation of ∼60 nucleotide precursor miRNAs (Denli et al., 2004; Kadener et al., 2009). To examine whether the change in expression of the processed miRNAs in the chimeras was due to altered Drosha processing, *in vitro* processing assays were performed with transcripts generated from the chimeras using the methodology described in our previous studies (Chawla and Sokol, 2014; Luhur et al., 2014; Chawla et al., 2016) (Figures 4D–F) (Supplementary Figures S4A–C). The rate of processing was examined by incubating the labeled transcripts with immunoprecipitated Drosha-Pasha complex (Supplementary Figure S4C). Both Pri-miR-100 and pri-miR-125 were processed less efficiently compared to pri-let-7. Approximately 0.5% of the wild type pri-miR-100 was processed within 60 min (Figure 4D) (Supplementary Figure S4A). In contrast 0% processing was observed for pri-miR-100Hlet-7B (Figure 4D) (Supplementary Figure S4A). Approximately 21% of pri-let-7 was processed within 30 min. The pri-let-7HmiR-100B chimera was processed much less efficiently with only 1.2% being processed in 30 min and no processing was observed with let-7HmiR-125B (Figure 4E) (Supplementary Figure S4B). The wild type pri-miR-125 transcript was processed much less efficiently with 0.2% being processed within 30 min. In contrast a significant increase in processing of pri-miR-125Hlet-7B was observed with 25% of the transcript being processed in the first 30 min and the processing of this transcript increased to 34% by 90 min (Figure 4F) (Supplementary Figure S4C). These data confirmed that the pri-let-7 stem-base enhanced processing of pri-miR-125 by Drosha.

To examine the expression of the three miRNAs *in vivo*, quantitative real time PCR was performed with total RNA extracted from transgenic lines expressing a single copy of the chimeric polycistronic transgenes under the control of the *let-7-C* Gal4 (Figures 4G,H). Consistent with the pri-miR-125Hlet-7B monocistronic transcript expression pattern, introducing this chimera in the UAS *let-7-C* cDNA (Constructs 3, 5, and 7) resulted in an increase in the levels of miR-125 (Figure 4H). In constructs where pri-miR-100Hlet-7B was introduced (Constructs 6 and 7), a significant decrease in miR-100 expression (Figure 4I) was detected.

**FIGURE 5:**
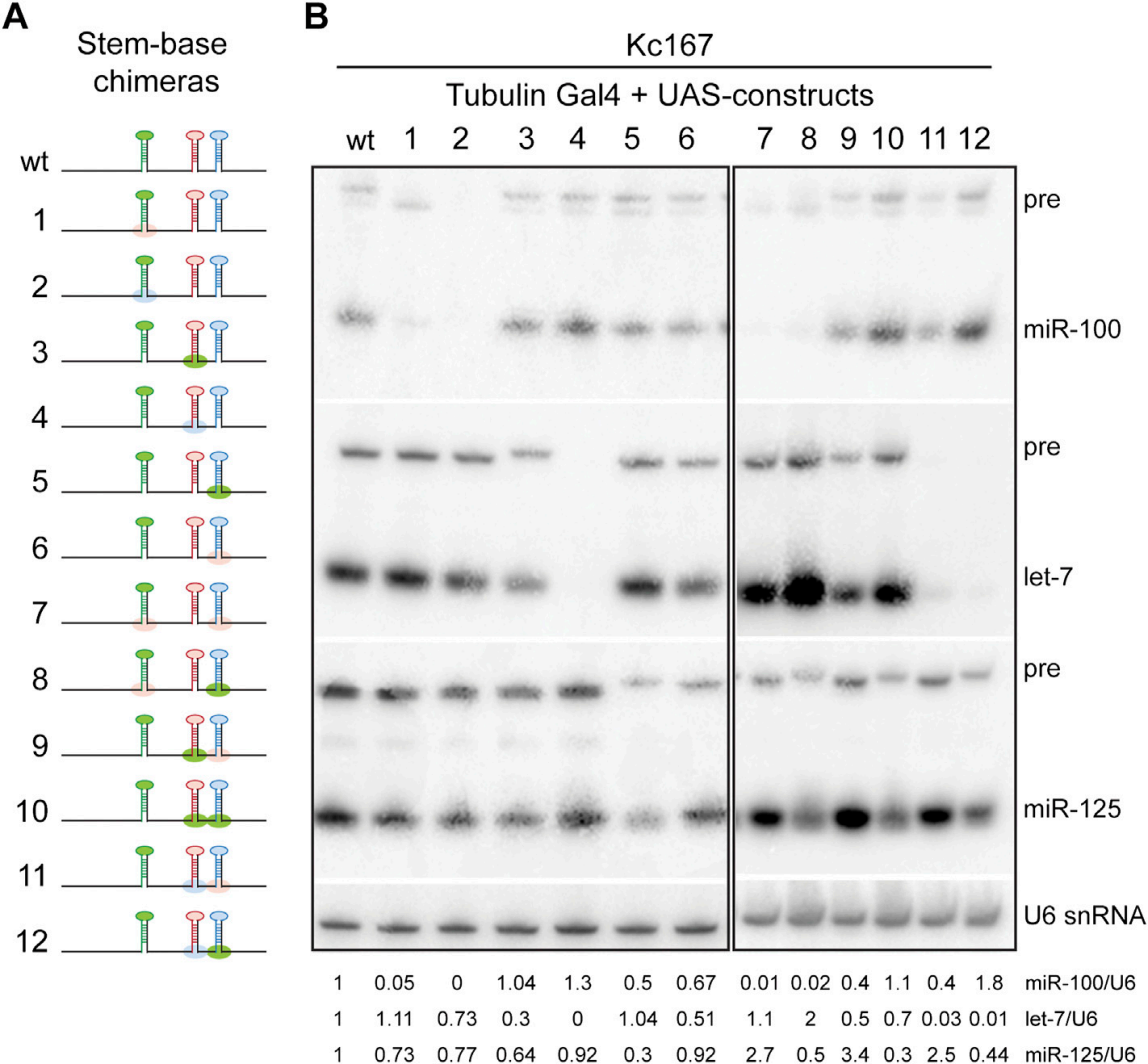
Northern blot analysis of stem-base chimeras of primary *let-7-C* transcript. **(A)** Schematic of the *UAS let-7-C* constructs for determining the effect of stem base in processing of *let-7-C* miRNAs. **(B)** Northern blot analysis of transfected Kc167 cells probed for *miR-100* (top panel), let-7 (middle panel) and miR-125 (bottom panel). Expression plasmids (UAS constructs) were co-transfected with Tubulin Gal4 plasmid and total RNA was extracted after 72 h of transfection. U6 snRNA was used as a control for normalization (lowermost panel) and the normalized levels of processed miRNAs are indicated below the gel.

The expression of the polycistronic wild type (synthetic i.e., where the wild type pri-miRNAs were reinserted after incorporating restriction sites) and stem-base chimeras was also examined by small RNA northern blots performed with total RNA extracted from Kc167 cells cotransfected with Tubulin Gal4 and UAS let-7-C cDNAs (Figures 5A,B). In addition to the expected changes in monocistronic pri-miRNAs, we also observed changes in the adjacent unmodified miRNAs, thus highlighting that the expression of the three miRNAs was dependent on the expression of adjacent miRNAs under conditions of overexpression. This confirmed that pri-miR-100 processing was dependent on the processing of pri-let-7 and pri-miR-125. Taken together these data suggest that substituting pri-miR-100 stem-base with either pri-let-7 or pri-miR-125 significantly reduces processing of the primary transcript. In contrast, pri-let-7 stem-base leads to efficient processing of both let-7 and miR-125. Moreover, increasing processing of pri-miR-125 lead to an increased repression of the canonical miR-125 sensor. Taken together, these data indicate that stem-bases of the primary hairpins are critical determinants of processing efficiency and are important for defining the structural features for precise Drosha cleavage.

### Terminal Loop (L) Region Determines Drosha and Dicer Processing and Expression of *let-7-C* miRNAs

**FIGURE 6:**
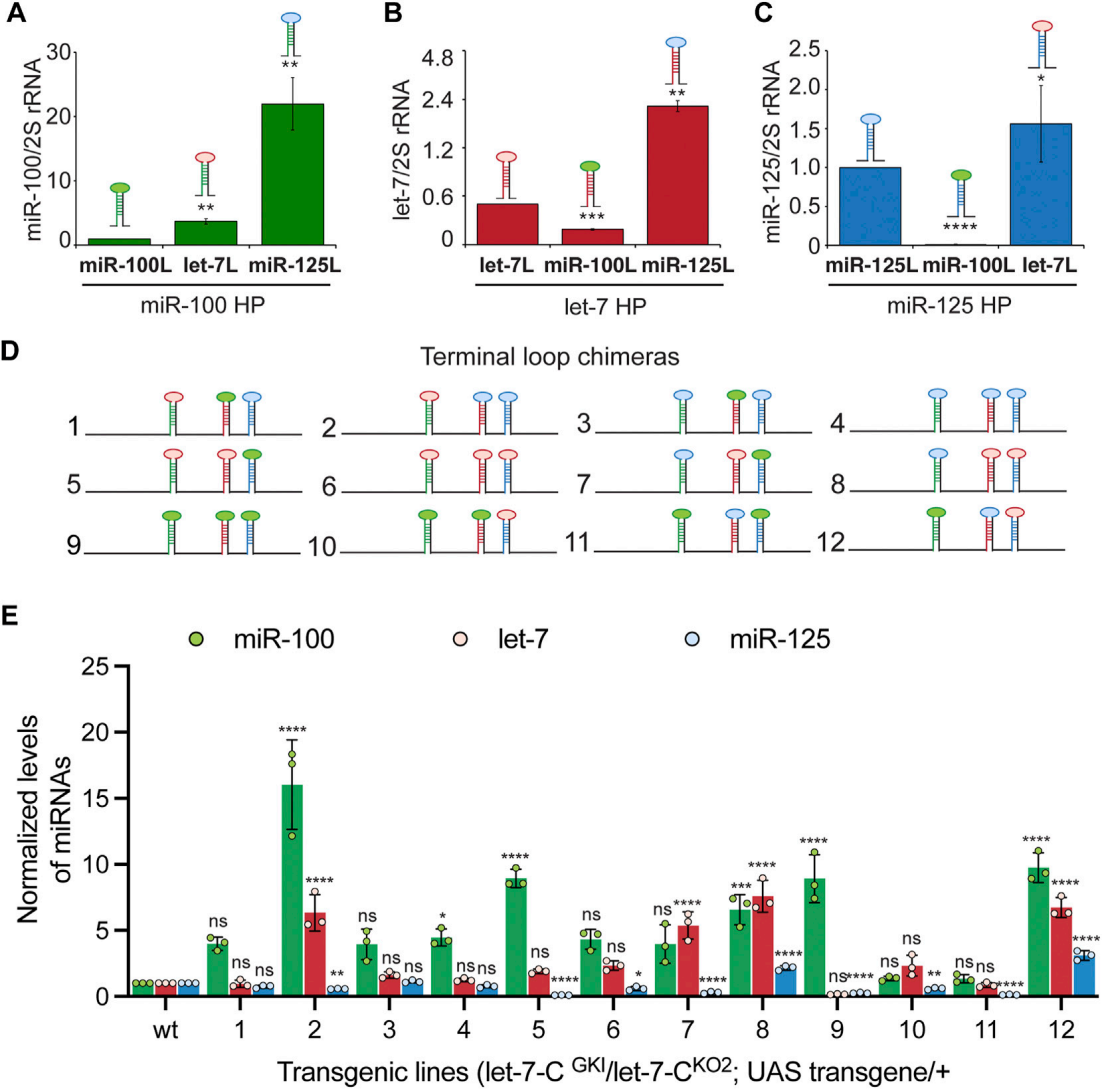
Terminal loops are critical determinants for expression of *let-7-Complex* miRNAs. **(A)** Expression of miR-100 in transgenic lines expressing *UAS pri*-*miR-100, UAS pri-miR-100Hlet-7TL, and UAS pri-miR-100HmiR-125TL* under the control of *let-7-C* Gal4 knock-in driver (*G4KI*) as determined by Taqman miRNA assays. Data are represented as mean ± SD, *n* = 3. *p*-value calculated by ordinary one-way ANOVA is 2.33E-04. **(B)** Expression of let-7 in transgenic lines expressing *UAS pri*-*let-7, UAS pri-let-7HmiR-100TL, and UAS pri-let-7HmiR-125TL* under the control of *let-7-C* Gal4 knock-in driver (*G4KI*) as determined by Taqman miRNA assays. Data are represented as mean ± SD, n = 3. *p*-value calculated by ordinary one-way ANOVA is 3.96E-08. **(C)** Expression of miR-125 in transgenic lines expressing *UAS pri-miR-125, UAS pri-miR-125HmiR-100TL, and UAS pri-miR-125Hlet-7TL* under the control of *let-7-C* Gal4 knock-in driver (*G4KI*) as determined by Taqman miRNA assays. Data are represented as mean ± SD, *n* = 3. *p*-value calculated by ordinary one-way ANOVA is 1.95E-03. **(D)** Schematic representation of UAS let-7-C chimeras that are described in panels **(E)**. **(E)** Expression of *miR-100* (green bar), *let-7* (red bar), and *miR-125* (blue bar) in transgenic lines expressing *UAS let-7-C* cDNA wild type and chimeras under the control of *let-7-C* Gal4 knock-in driver (*G4KI*) as determined by Taqman miRNA assays. Total RNA was extracted from adult flies that were in a trans heterozygote *let-7-C* null mutant of the following genotype: *let-7-C* ^*GKI*^/*let-7-C* ^*KO2*^ *; UAS transgene/+*. 2S rRNA was used as a control for normalization. Data are represented as mean ± SD, *n* = 3. *p*-value calculated by ordinary one-way ANOVA for miR-100 is 2.20E-12; for let-7 is 4.60E-14; and for miR-125 is < 1.00E-15. Adjusted *p*-values after applying Bonferroni’s correction are represented in the figure panel and we used an *α* level of 0.05 to assess statistical significance. Genotype of strains used: (6A) *w* ^*1118*^ *; let-7-C* ^*GKI*^ */let-7-C* ^*KO2*^ *, P{neoFRT}40A; {w+, UAS miR-100} VK00033/+; w* ^*1118*^ *; let-7-C* ^*GKI*^ */let-7-C* ^*KO2*^ *, P{neoFRT}40A; {w+, UAS miR-100Hlet-7TL} VK00033/+*; *w* ^*1118*^ *; let-7-C* ^*GKI*^ */let-7-C* ^*KO2*^ *, P{neoFRT}40A; {w+, UAS miR-100HmiR-125TL}VK00033/+* ;(6B) w^1118^; let-7-C^GKI^ / let-7-C^KO2^, P{neoFRT}^40A^; {w+, UAS let-7} VK00033/+; w^1118^; let-7-C^GKI^ / let-7-C^KO2^, P{neoFRT}^40A^; {w+, UAS let-7HmiR-100TL} VK00033/+; w^1118^; let-7-C^GKI^ / let-7-C^KO2^, P{neoFRT}^40A^; {w+, UAS let-7HmiR-125TL}VK00033/+; (6C) w^1118^; let-7-C^GKI^ / let-7-C^KO2^, P{neoFRT}^40A^; {w+, UAS miR-125} VK00033/+; w^1118^; let-7-C^GKI^ / let-7-C^KO2^, P{neoFRT}^40A^; {w+, UAS miR-125HmiR-100TL} VK00033/+; w^1118^; let-7-C^GKI^ / let-7-C^KO2^, P{neoFRT}^40A^; {w+, UAS miR-125Hlet-7TL}VK00033/+; (6K) *w* ^*1118*^ *; let-7-C* ^*GKI*^ */let-7-C* ^*KO2*^ *, P{neoFRT}40A; {w+, UAS let-7-C* ^*miR-100, let-7, miR-125*^ *} VK00033/+; w* ^*1118*^ *; let-7-C* ^*GKI*^ */let-7-C* ^*KO2*^ *, P{neoFRT}40A; {w+, UAS let-7-C* ^*miR-100let-7TL, let-7HmiR-100TL, miR-125*^ *} VK00033/+; w* ^*1118*^ *; let-7-C* ^*GKI*^ */let-7-C* ^*KO2*^ *, P{neoFRT}40A; {w+, UAS let-7-C* ^*miR-100let-7TL, let-7HmiR-125TL, miR-125*^ *} VK00033/+; w* ^*1118*^ *; let-7-C* ^*GKI*^ */let-7-C* ^*KO2*^ *, P{neoFRT}40A; {w+, UAS let-7-C* ^*miR-100HmiR-125TL, let-7HmiR-100TL, miR-125*^ *} VK00033/+*; *w* ^*1118*^ *; let-7-C* ^*GKI*^ */let-7-C* ^*KO2*^ *, P{neoFRT}40A; {w+, UAS let-7-C* ^*miR-100HmiR-125TL, let-7HmiR-125TL, miR-125*^ *} VK00033/+; w* ^*1118*^ *; let-7-C* ^*GKI*^ */let-7-C* ^*KO2*^ *, P{neoFRT}40A; {w+, UAS let-7-C* ^*miR-100Hlet-7TL, let-7, miR-125HmiR-100TL*^ *} VK00033/+; w* ^*1118*^ *; let-7-C* ^*GKI*^ */let-7-C* ^*KO2*^ *, P{neoFRT}40A; {w+, UAS let-7-C* ^*miR-100let-7TL, let-7, miR-125Hlet-7TL*^ *} VK00033/+; w* ^*1118*^ *; let-7-C* ^*GKI*^ */let-7-C* ^*KO2*^ *, P{neoFRT}40A; {w+, UAS let-7-C* ^*miR-100HmiR-125TL, let-7, miR-125HmiiR-100TL*^ *} VK00033/+; w* ^*1118*^ *; let-7-C* ^*GKI*^ */let-7-C* ^*KO2*^ *, P{neoFRT}40A; {w+, UAS let-7-C* ^*miR-100HmiR-125TL, let-7, miR-125Hle-7TL*^ *} VK00033/+; w* ^*1118*^ *; let-7-C* ^*GKI*^ */let-7-C* ^*KO2*^ *, P{neoFRT}40A; {w+, UAS let-7-C* ^*miR-100, let-7HmiR-100TL, miR-125HmiR-100TL*^ *} VK00033/+; w* ^*1118*^ *; let-7-C* ^*GKI*^ */let-7-C* ^*KO2*^ *, P{neoFRT}40A; {w+, UAS let-7-C* ^*miR-100, let-7HmiR-100TL, miR-125Hlet-7TL*^ *} VK00033/+; w* ^*1118*^ *; let-7-C* ^*GKI*^ */let-7-C* ^*KO2*^ *, P{neoFRT}40A; {w+, UAS let-7-C* ^*miR-100, let-7HmiR-125TL, miR-125HmiR-100TL*^ *} VK00033/+; w* ^*1118*^ *; let-7-C* ^*GKI*^ */let-7-C* ^*KO2*^ *, P{neoFRT}40A; {w+, UAS let-7-C* ^*miR-100, let-7HmiR-125TL, miR-125Hlet-7TL*^ *} VK00033/+.*

The terminal loops (L) of primary and precursor miRNAs function as binding sites for RNA binding proteins that can modulate miRNA biogenesis. Hence, we examined the contribution of these *cis*-acting elements in the expression and/or processing of pri-let-7-C miRNAs. To examine the expression of the Loop chimeras *in vivo*, we utilized Taqman miRNA real time PCR assay to quantitate the expression of the processed *let-7-C* miRNAs in 3-day old adult flies (Figures 6A–C). Substitution of pri-miR-100L with the pri-let-7L (3.6-fold/360% increase compared to wild type) or pri-miR-125L (22-fold/2200% increase compared to wild type) resulted in a significant increase in expression of miR-100 (Figure 6A). While replacing pri-let-7L with pri miR-100L (38% relative to wild type) resulted in a significant decrease in let-7 levels (Figure 6B), replacing pri-let-7L with pri-miR-125L resulted in a significant increase in processed let-7 (3.4-fold/343% increase relative to wild type). Substitution of pri-miR-125L with pri-miR-100L significantly reduced the levels of processed miR-125 (1.33% relative to wild type) and substitution with pri-let-7L resulted in 1.84-fold (184% increase relative to wild type) increase in the expression of miR-125 levels (Figure 6C).

**FIGURE 7:**
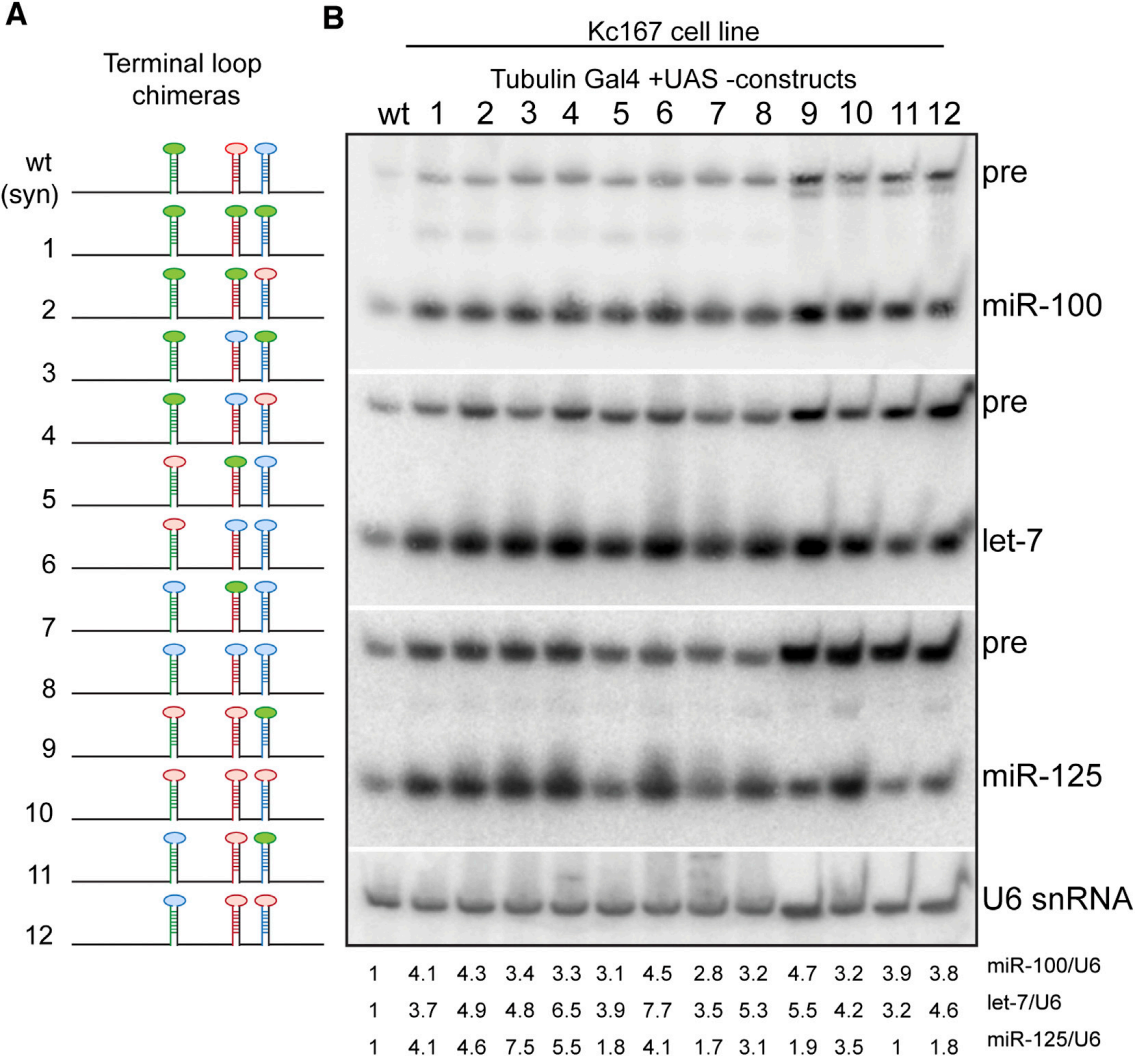
Expression of terminal loop chimeras of primary *let-7-C* transcript in Kc167 cell line. **(A)** Schematic of the *UAS let-7-C* constructs for determining the effect of terminal loop in processing of *let-7-C* miRNAs. **(B)** Northern blot analysis of transfected Kc167 cells probed for *miR-100* (top panel), let-7 (middle panel) and miR-125 (bottom panel). Expression plasmids (UAS constructs) were co-transfected with Tubulin Gal4 plasmid and total RNA was extracted after 72 h of transfection. U6 snRNA was used as a control for normalization (lowermost panel) and the normalized levels of processed miRNAs are indicated below the gel.

Next, we examined the expression of the three miRNAs in terminal loop chimeras in the context of the *let-7-C* cluster by RT-PCR in transgenic lines and small RNA northern blots in transfected Kc167 cell line (Figures 6D,E, 7A,B). To assess whether the terminal loops influenced expression of the miRNAs *in vivo*, the expression of the three miRNAs was examined in transgenic lines expressing a single copy of the transgene under the control of the *let-7-C* promoter (Figure 6D). Transgenic analysis revealed that the swapping of terminal loops between the primary hairpins resulted in changes in expression of the processed miRNAs and could be used as a strategy to fine tune the dosage of a miRNA in a context-dependent manner. However, the magnitude of the effects varied depending on the adjacent primary transcripts in the cluster. Substituting pri-miR-100L with pri-let-7L (Construct 2 and 5) or pri-miR-125L (Construct 4 and 8) resulted in an increased expression of miR-100 (Figure 6E). Similarly, substituting pri-let-7L with pri-miR-125L resulted in a significant increase in let-7 levels in lines expressing constructs 2 and 12 but not in lines expressing constructs 4 and 11 (Figure 6E). A significant increase in let-7 levels was also observed when prilet-7HmiR-125L harboring constructs were transfected in Kc167 cells (Constructs 3, 4, 6 and 8) (Figure 7B). An increase in processed miR-125 was detected in 2 of the 4 constructs that harbored pri-miR-125Hlet-7L (Constructs 8 and 12) (Figure 6E). An increase in the levels of processed miR-125 was also observed in northern blot analysis (Constructs 2, 4, 10 and 12) (Figure 7B). These differences in expression levels of the mature miRNAs highlight the interdependence of adjacent pri-miRNAs and offer an opportunity to design artificial chimeras to specifically alter expression of only one or two or all three miRNAs in the cluster. Future studies that identify the terminal loop binding proteins will likely aid in identifying therapeutics targets for modifying the expression of the miRNAs in different disease contexts. In summary, terminal loops play an important role in modulating the biogenesis of *let-7-C* miRNAs. Our analysis uncovered two configurations that resulted in higher expression of all three miRNAs in the cluster (Construct 8 and 12) (Figure 6E). Thus, altering the terminal loops of individual pri-miRNAs in this cluster may be considered as a plausible mechanism to modulate the miRNA biogenesis.

**FIGURE 8:**
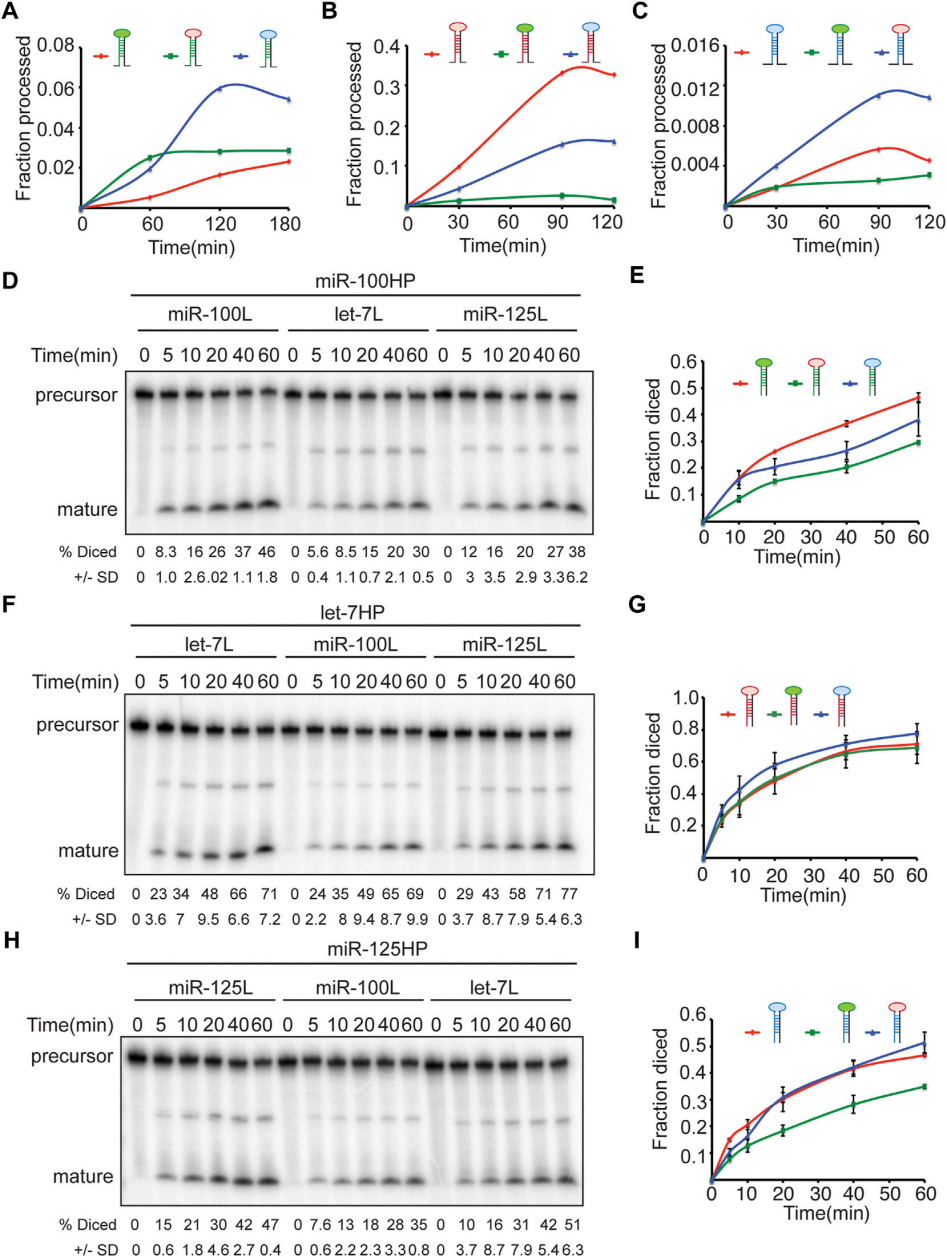
Terminal loops determine the kinetics of Drosha and Dicer processing of *let-7-C* miRNAs. **(A–C)** Chimeric and wild type transcripts were subjected to Drosha cleavage, and the fraction of processed pre-miRNA was calculated as the ratio of pre/pre+pri. Line graph depicting kinetics of processing of wild type (red line) and chimeric pri-miR-100 (blue and green) **(A)**, wild type (red line) and chimeric pri-let-7 (green and blue) **(B)**, and wild type (red line) and chimeric pri-miR-125 **(C)**. **(D–I)** Chimeric and wild type transcripts were subjected to Dicer cleavage, and the fraction of processed miRNA was calculated as the ratio of pro/pre+pro. **(D)** Pre-miR-100 was processed more efficiently than pre-miR-100Hlet-7L or pre-miR100HmiR-125L. Products were resolved by gel electrophoresis on a 10% polyacrylamide gel. **(E)** Line graph depicting kinetics of processing of wild type (red line) and chimeric pre-miR-100 (blue and green). **(F)** Pre-let-7HmiR-125L is diced more efficiently than pre-let-7 or pre-let-7HmiR-100L. Products were resolved by gel electrophoresis on a 10% polyacrylamide gel. **(G)** Wild type (red line) and chimeric pre-let-7 (green and blue). **(H)** Pre-miR-125 and pre-miR-125Hlet-7L are diced more efficiently than pre-miR-125HmiR-100L. **(I)** Line graph depicting kinetics of processing of wild type (red line) and chimeric pre-miR-125 (blue and green).

The terminal loop forms an integral part of both primary as well as precursor miRNAs and has been shown to be recognized by RNA binding proteins that can modulate Drosha and Dicer processing (Treiber et al., 2019), hence, we examined the kinetics of processing of the terminal loop chimeras by Drosha and Dicer (Figure 8) (Supplementary Figures S4A–D). We first quantitated the rate of generation of pre-miRNAs from pri-miRNA transcripts by Drosha-Pasha complex (Figures 8A–C) (Supplementary Figures S4A–C). The rates of processing of the transcripts were examined by incubating with immunoprecipitated Drosha-Pasha complexes as described in our previous studies (Chawla and Sokol, 2014; Chawla et al., 2016). Substituting the terminal loop of pri-miR-100 with the terminal loop of either pri-let-7 or pri-miR-125 resulted in an increase in Drosha processing (Figure 8A). While 0.5% of the wild type pri-miR-100 was processed by 60 min, 2.5% of pri-miR-100Hlet-7L and 1.98% of pri-miR-100HmiR-125L was cleaved by Drosha. Incubation with Drosha-Pasha for 120 min increased the percentage of precursor to 1.6%, 2.8% and 5.9% for pri-miR-100, pri-miR-100Hlet-7L and pri-miR-100HmiR-125L, respectively (Figure 8A). Drosha processing of unmodified pri-let-7 and terminal loop chimeras indicated that unmodified pri-let-7 was processed most efficiently by Drosha. While 9.8% of the unmodified let-7 primary transcript was processed within 30 min, only 1.4% of pri-let-7miR-100L and 4.3% of pri-let-7miR-125L was cleaved by Drosha. Incubation with Drosha-Pasha complex for 90 min increased the percentage of precursor to 33%, 2.6% and 15% for pri-let-7, pri-let-7miR-100L, and pri-let-7miR-125L, respectively (Figure 8B). Substituting the terminal loop of pri-miR-125 with the terminal loop of pri-let-7 resulted in an increase in Drosha processing (Figure 8B; Supplementary Figure S4B). While 0.18% of the wild type pri miR-125 was processed within 30 min, 0.4% of pri-miR-125Hlet-7L was cleaved by Drosha. Incubation with Drosha-Pasha for 90 min increased the percentage of precursor to 0.5% and 1.1% for pri-miR-125 and pri-miR-125Hlet-7L, respectively (Figure 8B). The pri-miR-125HmiR-100L was cleaved less efficiently with 0.25% of precursor being generated in 90 min (Figure 8C) (Supplementary Figure 4C). Dicer-1 processing of the wild type and terminal loop chimeras was examined by performing *in vitro* processing assays with Flag-tagged Dicer-1 that was purified from Kc167 cells as described in our previous study (Chawla et al., 2016). In these assays, pre-miR-100 was diced more efficiently as compared to pre-miR-100HmiR-125L and pre-miR-100Hlet-7L (Figures 8D,E). 16.2% pre-miR-100, 15.7% of pre-miR-100HmiR-15L and 8.5% of pre-miR-100Hlet-7L was cleaved within 10 min of incubation with Dicer 1 (Figures 8D,E). After 60 min, the percentage diced increased to 46%, 38% and 29.7% for pri-miR-100, pri-miR-100HmiR-125L and pri-miR-100Hlet-7L, respectively (Figure 8D). For pre-let-7 wild type and terminal loop chimeras, pre-let-7HmiR-125L displayed the most efficient kinetics of processing compared to the wild type pre-let-7 and pre-let-7HmiR-100L (Figures 8F,G). Within 10 min of incubation with Dicer 1, 22.6% of pre-let-7, 24% of pre-let-7HmiR-100L and 29% of pre-let-7HmiR-125L were processed. After 60 min 71% of pre-let-7, 69% of pre-let-7HmiR-100L and 78% of pre-let-7HmiR-125L were processed (Figure 8F). Pre-miR-125 and pre-miR-125hlet-7L were processed at comparable levels by Dicer and their kinetics of processing was much higher than that or pre-miR-125HmiR-100L (Figures 8H,I). Within 10 min of incubation, 15% of pre-miR-125, 10.4% of pre-miR-125Hlet-7L and 7.6% of pre-miR-125HmiR-100L were diced. After 60 min of incubation with Dicer 1, the percentage diced was 47%, 51% and 35% for pre-miR-125, pre-miR-125Hlet-7L and pre-miR-125HmiR-100L, respectively (Figures 8H,I). Thus, pre-miR-125Hlet-7L was more efficiently processed by both Drosha and Dicer as compared to the wild type and pre-miR-125HmiR-100L. Since terminal loops are often recognized by RNA binding proteins that modulate the activity of Drosha and/or Dicer machinery. Identification and characterization of the auxiliary factors can also be employed for modulating the miRNA activity.

## Discussion

Approximately 50% of miRNA loci in *Drosophila melanogaster* reside in close proximity in the genome to form clusters that are transcribed together. These clustered miRNAs are predominantly co-expressed and regulate functionally related target mRNAs (Biemar et al., 2005; Ruby et al., 2007; Kim et al., 2009; Chawla and Sokol, 2012). Since, these clusters are transcribed as a single primary transcript, fine-tuning of the downstream effector pathways is achieved by post-transcriptional regulatory mechanisms that determine the processing efficiency (Heo et al., 2008; Choudhury and Michlewski, 2012; Chawla and Sokol, 2014; Kooshapur et al., 2018). The cis-acting sequence and structural features of the primary miRNA that interact with the processing machinery are critical determinants of the processing efficiency of the primary and precursor miRNAs (Han et al., 2006; Auyeung et al., 2013; Fang and Bartel, 2015). More specifically, the primary sequence in the single stranded RNA (ssRNA) in the basal region and terminal loop RNA enhance recognition by the Microprocessor (Auyeung et al., 2013). Other studies indicate that the basal ssRNA and/or terminal loop and other structural/sequence features of a pri-miRNA such as the stem and internal loops are required for recognition and processing (Zeng and Cullen, 2005; Han et al., 2006; Zeng and Cullen, 2006; Zhang and Zeng, 2010; Feng et al., 2011; Feng et al., 2012; Ma et al., 2013; Burke et al., 2014; Fang and Bartel, 2015; Kwon et al., 2019; Zhang et al., 2021). Some of the features predicted by Mfold that may contribute to differential processing of the monocistronic pri-miRNAs and their chimeras are 1) a larger terminal loop of pri-let-7 resulting in efficient processing of wild type pri-let-7; 2) optimum length of pri-let-7 stem (22bp) to direct Drosha cleavage; 3) smaller terminal loop in pri-miR-125 resulting in decreased efficiency of Drosha processing; 4) multiple internal loops in pri miR-125; 5) absence of ssRNA extensions and presence of strong secondary structure in pri-miR-100Hlet-7B, pri-miR-100HmiR-125B and pri-let-7HmiR-125B resulting in decreased Drosha cleavage (Supplementary Figures S2, S3). Our findings that the kinetics of Drosha processing correlate with the expression of the processed miRNA (let-7 > miR-125 > miR-100) are consistent with studies that have shown that Drosha processing largely determine genome wide differential expression of a miRNA (Feng et al., 2011; Conrad et al., 2015; Zhang et al., 2021).

However, this study and our previous analysis of the *let-7-C* polycistronic transcript also hint at the existence of other post-transcriptional mechanisms that may function to fine-tune expression of the conserved miRNAs within this cluster (Chawla and Sokol, 2014). In this study we have investigated the contribution of position, terminal loops and stem-base of pri-miR-100, pri-let-7 and pri-miR-125. We found that changing the position of the miR-100 stem loop from first to the third position resulted in a significantly reduced expression and activity of miR-100 (Figure 1C). However, changing the position of pri-let-7 or pri-miR-125 did not significantly influence the functional activity of the more efficiently processed let-7 and miR-125 despite an increase in expression levels *in vivo*.

Biogenesis of suboptimal miRNAs is enhanced when they are present adjacent to neighboring optimally processed miRNAs (Truscott et al., 2016; Park et al., 2021). Based on the annotation on miRBase release 22.1, the number of reads from 49 experiments are 14632 for miR-100-5p, 895953 for let-7-5p and 97490 for miR-125-5p, respectively (Aravin et al., 2003; Lai et al., 2003; Kozomara and Griffiths-Jones, 2011; Kozomara and Griffiths-Jones, 2014; Kozomara et al., 2019). In addition, northern blot analysis of the three *let-7-C* miRNAs have also revealed a difference in expression levels (Sempere et al., 2003; Chawla and Sokol, 2012). These data indicated that miR-100 is processed less efficiently as compared to mir-125 and let-7. Hence, we examined the effect of deleting pri-miR-100, pri-let-7 and pri-miR-125 on expression and activity of the miRNAs processed from the adjacent stem loops (Figure 2). Deleting pri-let-7 significantly reduced the expression and activity of both miR-100 and miR-125 (Figures 2B–D). These expression data are consistent with our previous analysis with genomic rescue transgenes that included genomic regulatory elements that likely provided a more stringent control of expression and processing of the miRNA stem loops in the cluster (Chawla et al., 2016) and indicate that the clustering of pri-miR-100 and pri-mir-125 with pri-let-7 enhances the processing and functional activity of miR-100 and miR-125. However, it is worth mentioning that a similar analysis, showed that deletion of both miR-100 and let-7 and not let-7 alone resulted in a decrease in miR-125 (Truscott et al., 2016). The differences in results could be attributed to the differences in constructs, cell line and the time points (48 h vs. 72 h).

One of the striking results from our analysis with stem base chimeras was the significant increase in miR-125 in pri-miR-125Hlet-7B chimeric hairpins (Figures 3H, 4H, 5B). The increased expression of mature miR-125 in the chimeric hairpin lead to a concomitant increase in the fold repression of the miR-125 sensor (Figure 3I). The increased expression of pri-miR-125Hlet-7B was also observed in the polycistron (Figures 4H, 5B). In contrast, a significant decrease in miR-100 was observed in pri-miR-100Hlet-7B chimeric hairpins (Figures 3B, 4A, 5B). Thus, highlighting that the structural changes introduced by the insertion of the same sequence (let-7B) resulted in the difference in Drosha processing of pri-miR-100Hlet-7B and pri-miR-125Hlet-7B and a consequent change in the processed miRNA levels. The band corresponding to the pre-miR-100Hlet-7B migrated a little faster than the pre-miR-100 generated from the wildtype construct, thus indicating an alteration in the Drosha cleavage site and the possibility of generation of an isomiR with altered function (Figure 5B, construct 1, 7 and 8). Previous studies have highlighted the importance of alternate Drosha cleavage of pri-miRNAs encoded by the human pri-miR-9 family and generation of isomiRs of miR-9 that altered the selection of several reporters (Tan et al., 2014; Bofill-De Ros et al., 2019). The importance of Drosha cleavage fidelity in altering the function of a miRNA has been demonstrated by studies that identified sequence motifs that contributed to the efficiency of processing (Auyeung et al., 2013; Fang and Bartel, 2015) and by changing the distance between the expected cleavage site, the basal junction and the apical junction of a pri-miRNA (Ma et al., 2013; Burke et al., 2014; Roden et al., 2017). While several studies have highlighted the importance of Drosha cleavage in dictating mature miRNA function, the mechanism by which Drosha cleavage site is determined remains largely unknown (Bofill-De Ros et al., 2019). It has also been proposed that during evolution, Drosha cleavage at alternative sites has been selected to allow the formation of newer miRNAs (Ruby et al., 2007; Berezikov, 2011; Bofill-De Ros et al., 2019). Taken together our expression analysis together with Mfold predictions and the data from other studies indicate that Drosha cleavage is more critical than Dicer cleavage as the former determines the efficiency of the latter, with Dicer cleavage being guided by the ends of the pre-miRNA (MacRae et al., 2007; Park et al., 2021). Overall, these data indicate that the hairpin structure and not sequence per se is more critical for determining the efficiency and fidelity of Drosha cleavage.

Terminal loops and RNA binding proteins that recognize the sequences in the terminal loops form an important layer of regulation of miRNA biogenesis (Treiber et al., 2017; Kooshapur et al., 2018; Treiber et al., 2019). Hence, we examined the contribution of the terminal loops of pri-miR-100, pri-let-7 and pri-miR-125, by generating chimeric constructs (Figures 3, 6–8). Our analysis revealed that the terminal loop sequence/structure influenced kinetics of processing by Drosha and Dicer in a variable manner. The terminal loop that enhanced Drosha processing significantly, did not result in a similar effect on Dicer processing. For example, primiR-100HmiR-125L and pri-miR-100Hlet-7L were processed more efficiently by Drosha when compared to the wild type pri-miR-100 (Compare Figures 8A,D,E). However, the wild type pri-miR-100 was processed more efficiently by Dicer as compared to either of the two chimeras. However, transgenic lines expressing the pri-miR-100Hlet-7L or pri-miR-100HmiR-125L chimeras expressed significantly higher levels of miR-100 (Figure 6A). Taken together, these data have opened the possibility of existence of RNA binding proteins that recognize terminal loop sequences and modulate miRNA biogenesis in a context-dependent manner.

In conclusion, our data has uncovered the importance of structural determinants such as the terminal loops in differential processing of *let-7-C* miRNAs. Future studies focused on proteomic, biochemical, and structural approaches will likely aid in the identification of RNA-binding proteins that bind and regulate processing of this and other conserved clusters under different contexts. Understanding how the different pools of RNA binding proteins influence processing of conserved clusters of miRNAs in a cell type specific manner will provide insights on how different biological processes are regulated by miRNAs.

## Data Availability

The original contributions presented in the study are included in the article/Supplementary Material, further inquiries can be directed to the corresponding author.

## Supplementary Material

Supplementary Figure S1 Scheme for generation of strains used in Figures 1–5. The genotype of the flies that were analyzed (F12) were trans-heterozygous for two different *let-7-C* null alleles (pink and yellow), ensuring that phenotypes were not due to recessive mutations on either *let-7-C* mutant chromosome. In addition, third chromosomes that contained differing rescuing transgenes (light blue) were derived in parallel from the same population of flies. Finally, all flies had a common X-chromosome (green), derived from an isogenized stock. (**S1-1**) All UAS transgenes, including the wildtype UAS transgene as well as the other versions, were injected into embryos from the same population of BL#24871. Resulting progeny were backcrossed twice to BL#3703 in order to select and balance *white +* transformants (F1) Single transformants were subsequently backcrossed to an isogenized version of BL#3703 three times (F2-F4) in order to make balanced stocks with isogenized X chromosomes (F6). (**S1-2**) Stocks with differing rescuing transgenes were crossed to the same population of a stock that contained the *let-7-C* ^*KO2*^ chromosome, an isogenized X chromosome, and two 3rd chromosome balancers. The *let-7-C* ^*KO2*^ stock used in F7 was generated in a similar fashion as the rescuing transgenes stocks, by backcrossing three times to an isogenized version of BL#3703. Resulting stocks (F8) had common X (green), 2nd (pink) and 3^rd^ (blue) chromosomes and were used in F13 to generate the experimental strains. (**S1-3**) A second *let-7-C* allele, *let-7-C* ^*GKI*^, was prepared by outcrossing twice to an isogenized stock, and then crossed to an isogenized stock containing a T (2:3) CyO-TM6B compound chromosome. The *let-7-C* allele was selected based on mini-white, and the T (2:3) CyO-TM6B balancer was selected based on the dominant Humoral marker. The resulting stock with a fixed second and third chromosome was amplified and used as the source for all virgins in the crosses that yielded the flies for analysis. (**S1-4)** Flies for analysis were generated by crossing virgins of the stock generated in F12 with males of stocks generated in F8 that harbored differing rescuing transgenes.

Supplementary Figure S2 Mfold predicted structures of the primary let-7-C miRNA wild type and chimeric hairpins. **(A–E)** Predicted secondary structures of pri-miR-100 wild type **(A)**, pri-miR-100Hlet-7L **(B)**, pri-miR-100HmiR-125L **(C)**, pri miR-100Hlet-7B **(D)**, and pri-miR-100HmiR-125B **(E)**. **(F–J)** Predicted secondary structures of pri-let-7 wild type **(F)**, pri-let-7HmiR-100L **(G)**, pri-let-7HmiR-125L **(H)**, pri-let-7HmiR-100B **(I)**, and pri-let-7HmiR-125B **(J)**. **(K–O)** Predicted secondary structures of pri-miR-125 wild type **(K)**, pri-miR-125HmiR-100L **(L)**, pri-miR-125Hlet-7L **(M)**, pri-miR-125HmiR-100B **(N)**, and pri-miR-125Hlet-7B **(O)**. In cases where more than one structure was predicted by the software, only the most structures that were most thermodynamically stable have been represented in the figure.

Supplementary Figure S3 Mfold predicted structures of the precursor let-7-C miRNA wild type and chimeric hairpins. **(A–C)** Predicted secondary structures of pre-miR-100 wild type **(A)**, pre-miR-100Hlet-7L **(B)**, and pre-miR-100HmiR-125L **(C)**. **(D–F)** Predicted secondary structures of pre-let-7 wild type **(D)**, pre-let-7HmiR-100L **(E)**, and pre-let-7HmiR-125L **(F)**. **(G–I)** Predicted secondary structures of pre-miR-125 wild type **(D)**, pre-miR-125HmiR-100L **(E)**, and pre-miR-125Hlet-7L **(F)**.

Supplementary Figure S4
*In vitro* Drosha processing assays with primary let-7-C miRNAs wild type and chimeric hairpins. **(A–C)** *In vitro* processing of Pri-miR-100 wild type/chimeric transcripts **(A)**, pri-let-7 wildtype/chimeric transcripts **(B)** and, pri-miR-125 wild type/chimeric transcripts **(C)** was performed with purified Drosha-Pasha complex. The primary transcript (pri), 5′ flank (5′F), 3′ flank (3′F) and precursor (pre) are indicated on the right of the gels. Quantitation of the fraction processed is calculated as precursor/primary +5′F+3′F+precursor is represented in the line graphs in Figures 4, 8. **(D)** Western blot analysis of purified Flag-tagged Drosha-Pasha (top panel) used in the Drosha processing assays. The bottom panel is a flag-tagged control protein.
