# Supplementary material for: Molecular Dissection of a Conserved Cluster of miRNAs Identifies Critical Structural Determinants That Mediate Differential Processing
Source: Front Cell Dev Biol. 2022 Jun 17;10:909212. doi: 10.3389/fcell.2022.909212 (PMC9247461; doi:10.3389/fcell.2022.909212)
Supplement: Supplementary file 1 [file Table1.DOCX]

**Supplementary Table 1. Sequences of the primary and precursor transcripts described in the study**

| **Name** | **Sequence** |
| --- | --- |
| Pri-miR-100  (146 nt) | acuugaaauggugcauacuuacauauggaccauuaacagaaacccguaaauccgaacuugugcuguuuuauaucuguuacaagaccggcauuaugggagucugucaaugcaaacaacugguuuuuggcaacaaaaucaaugacaaa |
| Pri-miR-100H  let-7B  (136 nt) | aaaccaccuagcaaaaaggacuacaccaaggaccuuuuucucucuggcaagaaacccguaaauccgaacuugugcuguuuuauaucuguuacaagaccggcauuauggguuugcuugacuacaagccgcauuugau |
| Pri-miR-100H  miR-125B  (139 nt) | aauauuggcauuggugacaugugcaaauguuuguauggcuggaaacccguaaauccgaacuugugcuguuuuauaucuguuacaagaccggcauuauggguggacgcaaacuugcugauguuaguaaaaaauaaggcaa |
| Pri-miR-100Hlet-7L  (144 nt) | acuugaaauggugcauacuuacauauggaccauuaacagaaacccguaaauccgaacuugugguaauuacacaucauacaagaccggcauuaugggagucugucaaugcaaacaacugguuuuuggcaacaaaaucaaugacaa |
| Pri-miR-100H miR-125L  (146 nt) | acuugaaauggugcauacuuacauauggaccauuaacagaaacccguaaauccgaacuugugcuuuuaauaccaguuuacaagaccggcauuaugggagucugucaaugcaaacaacugguuuuuggcaacaaaaucaaugacaaa |
| Pre-miR-100  (61 nt) | aacccguaaauccgaacuugugcuguuuuauaucuguuacaagaccggcauuaugggaguc |
| Pre-miR-100Hlet-7L (60 nt) | aacccguaaauccgaacuugugguaauuacacaucauacaagaccggcauuaugggaguc |
| Pre-miR-100H miR-125L (61 nt) | aacccguaaauccgaacuugugcuuuuaauaccaguuuacaagaccggcauuaugggaguc |
| miR-100 5p (22 nt) | aacccguaaauccgaacuugug |
| Pri-let-7 (138 nt) | aaaccaccuagcaaaaaggacuacaccaaggaccuuuuucucucuggcaaauugagguaguagguuguauaguaguaauuacacaucauacuauacaaugugcuagcuuucuuugcuugacuacaagccgcauuugau |
| Pri-let-7HmiR100B  (148 nt) | acuugaaauggugcauacuuacauauggaccauuaacagaugagguaguagguuguauaguaguaauuacacaucauacuauacaaugugcuagcuuucagucugucaaugcaaacaacugguuuuuggcaacaaaaucaaugacaaa |
| Pri-let-7Hmir125B  (141 nt) | aauauuggcauuggugacaugugcaaauguuuguauggcugauugagguaguagguuguauaguaguaauuacacaucauacuauacaaugugcuagcuuucuggacgcaaacuugcugauguuaguaaaaaauaaggcaa |
| Pri-let-7H miR100L  (138 nt) | aaccaccuagcaaaaaggacuacaccaaggaccuuuuucucucuggcaaauugagguaguagguuguauaguacuguuuuauaucuguuacuauacaaugugcuagcuuucuuugcuugacuacaagccgcauuugau |
| Pri-let-7 H mir125L  (139 nt) | aaaccaccuagcaaaaaggacuacaccaaggaccuuuuucucucuggcaaauugagguaguagguuguauaguacuuuuaauaccaguuuacuauacaaugugcuagcuuucuuugcuugacuacaagccgcauuugau |
| Pre-let-7 (61 nt) | uugagguaguagguuguauaguaguaauuacacaucauacuauacaaugugcuagcuuucu |
| Pre-let-7H  miR 100L (62 nt) | uugagguaguagguuguauaguacuguuuuauaucuguuacuauacaaugugcuagcuuucu |
| Pre-let-7H mir125L  (62 nt) | uugagguaguagguuguauaguacuuuuaauaccaguuuacuauacaaugugcuagcuuucu |
| let-7 5p (21 nt) | ugagguaguagguuguauagu |
| Pri-miR-125  (142 nt) | aauauuggcauuggugacaugugcaaauguuuguauggcugauucccugagacccuaacuugugacuuuuaauaccaguuucacaaguuuugaucuccgguauuggacgcaaacuugcugauguuaguaaaaaauaaggcaa |
| Pri-miR-125H miR100B  (149 nt) | acuugaaauggugcauacuuacauauggaccauuaacagaucccugagacccuaacuugugacuuuuaauaccaguuucacaaguuuugaucuccgguauagucugucaaugcaaacaacugguuuuuggcaacaaaaucaaugacaaa |
| Pri-miR-125H let-7B For  (139 nt) | aaaccaccuagcaaaaaggacuacaccaaggaccuuuuucucucuggcaaauucccugagacccuaacuugugacuuuuaauaccaguuucacaaguuuugaucuccgguauuuugcuugacuacaagccgcauuugau |
| Pri-miR125H miR100L For  (142 nt) | aauauuggcauuggugacaugugcaaauguuuguauggcugauucccugagacccuaacuugugacuguuuuauaucuguucacaaguuuugaucuccgguauuggacgcaaacuugcugauguuaguaaaaaauaaggcaa |
| Pri-miR125H let-7L For  (142 nt) | aauauuggcauuggugacaugugcaaauguuuguauggcugauucccugagacccuaacuugugaguaauuacacaucaucacaaguuuugaucuccgguauuggacgcaaacuugcugauguuaguaaaaaauaaggcaa |
| Pre-miR-125  (60 nt) | ucccugagacccuaacuugugacuuuuaauaccaguuucacaaguuuugaucuccgguau |
| Pre-miR125H miR100L (60 nt) | ucccugagacccuaacuugugacuguuuuauaucuguucacaaguuuugaucuccgguau |
| Pre-miR125H let-7L (59 nt) | ucccugagacccuaacuugugaguaauuacacaucaucacaaguuuugaucuccgguau |
| miR-125 5p (22 nt) | ucccugagacccuaacuuguga |
