# Supplementary figures and images for: Molecular Dissection of a Conserved Cluster of miRNAs Identifies Critical Structural Determinants That Mediate Differential Processing
Source: Front Cell Dev Biol. 2022 Jun 17;10:909212. doi: 10.3389/fcell.2022.909212 (PMC9247461; doi:10.3389/fcell.2022.909212)

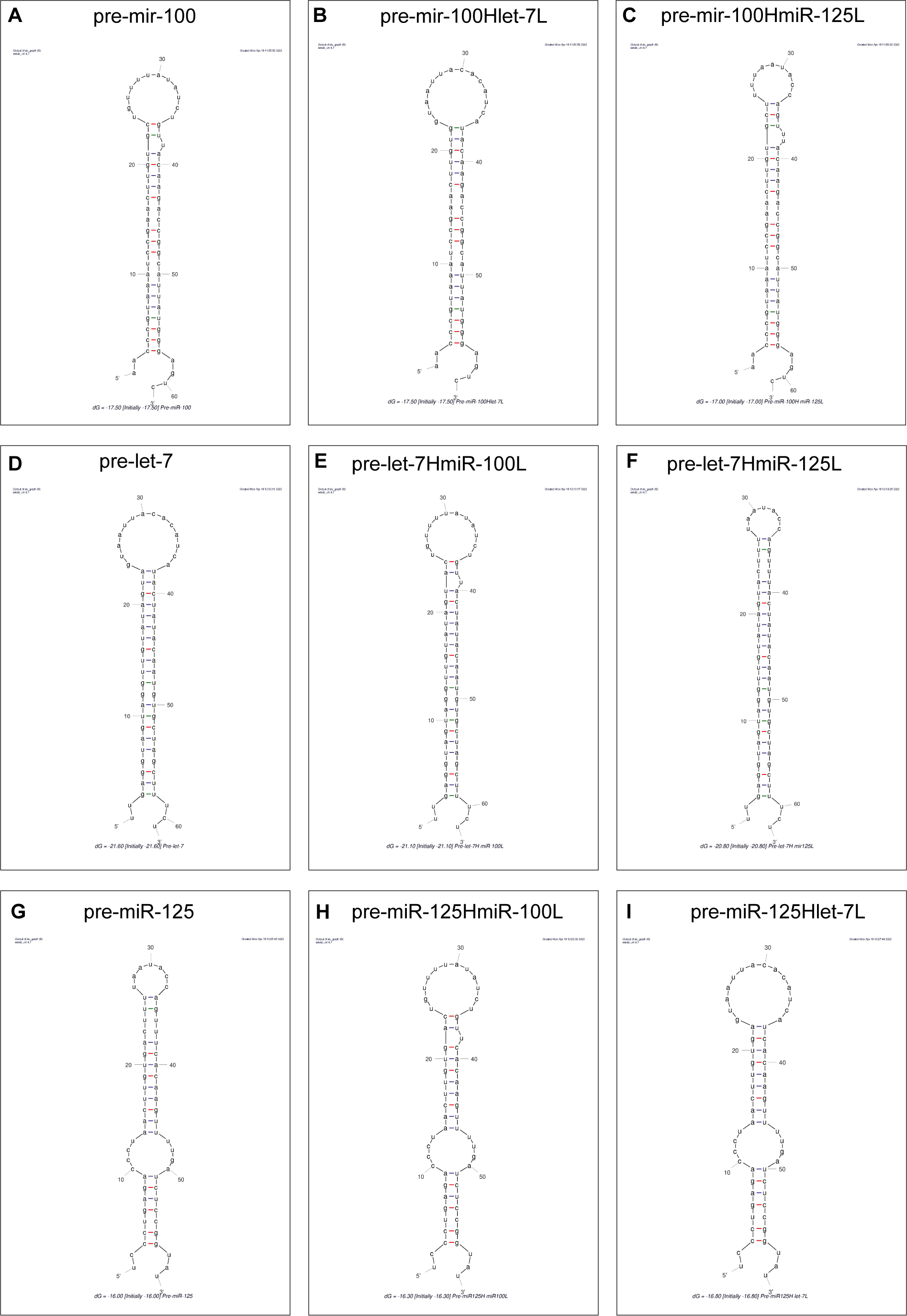

Supplement: Supplementary file 2 [file Image3.TIF]

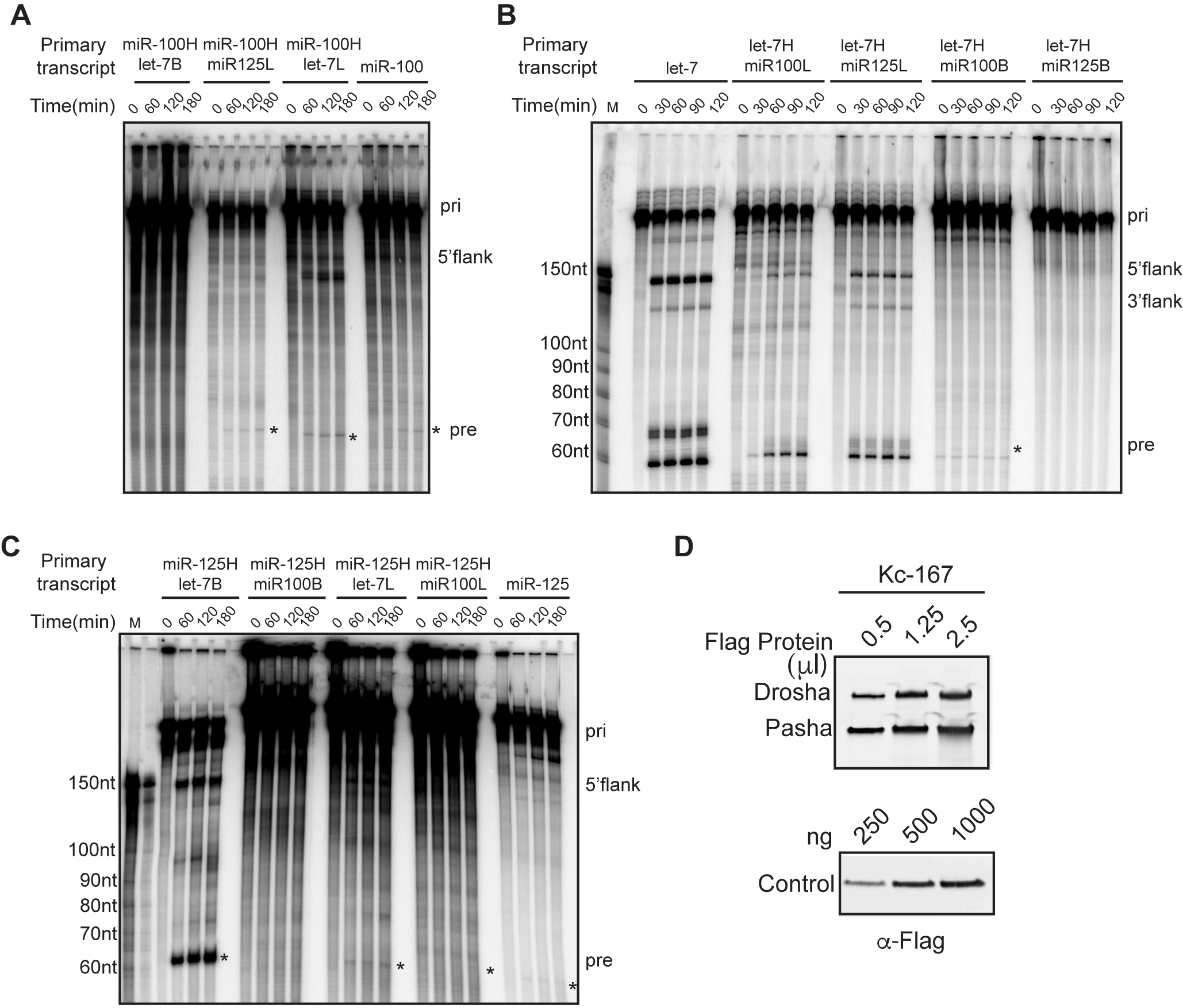

Supplement: Supplementary file 3 [file Image4.TIF]

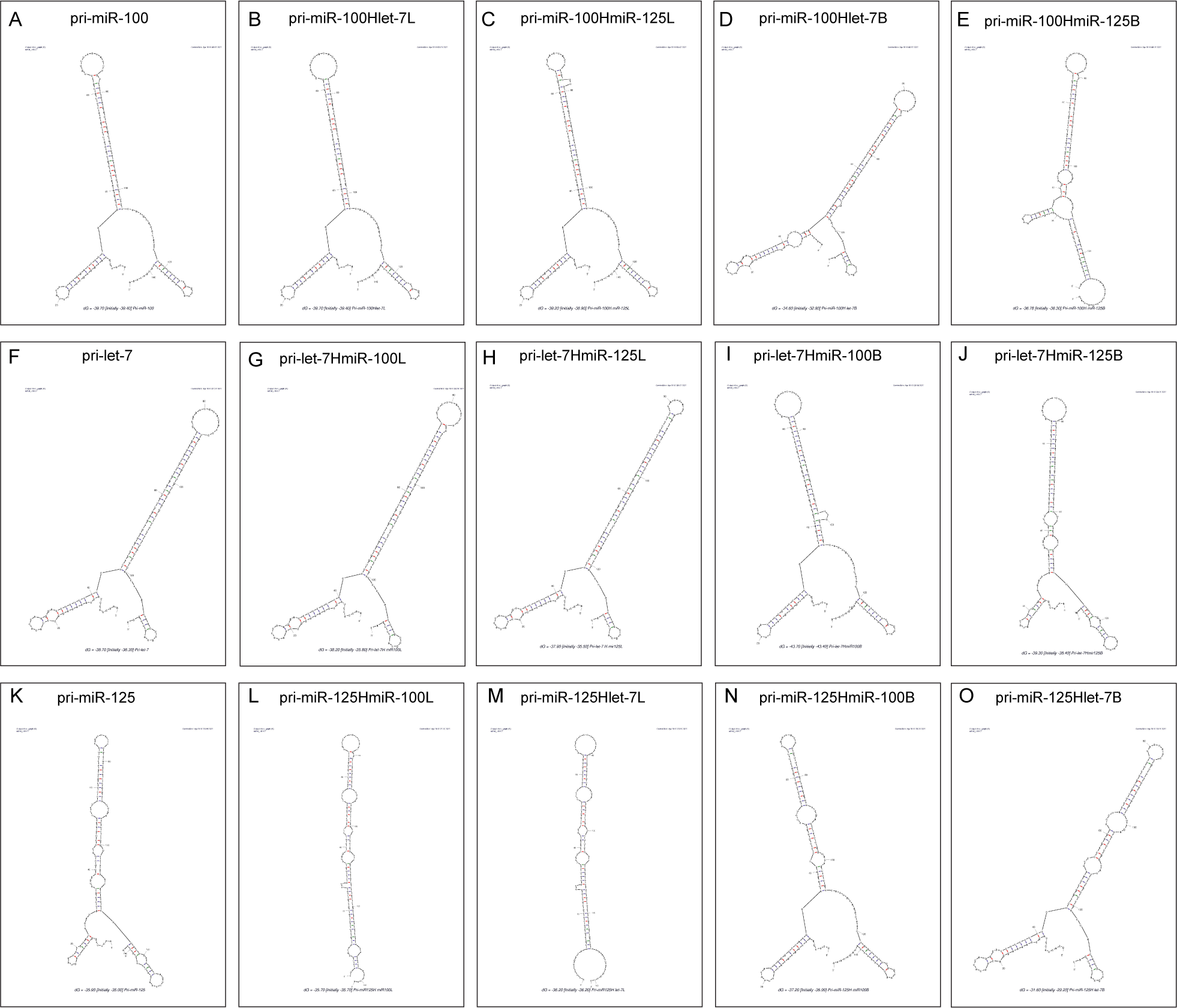

Supplement: Supplementary file 4 [file Image2.TIF]

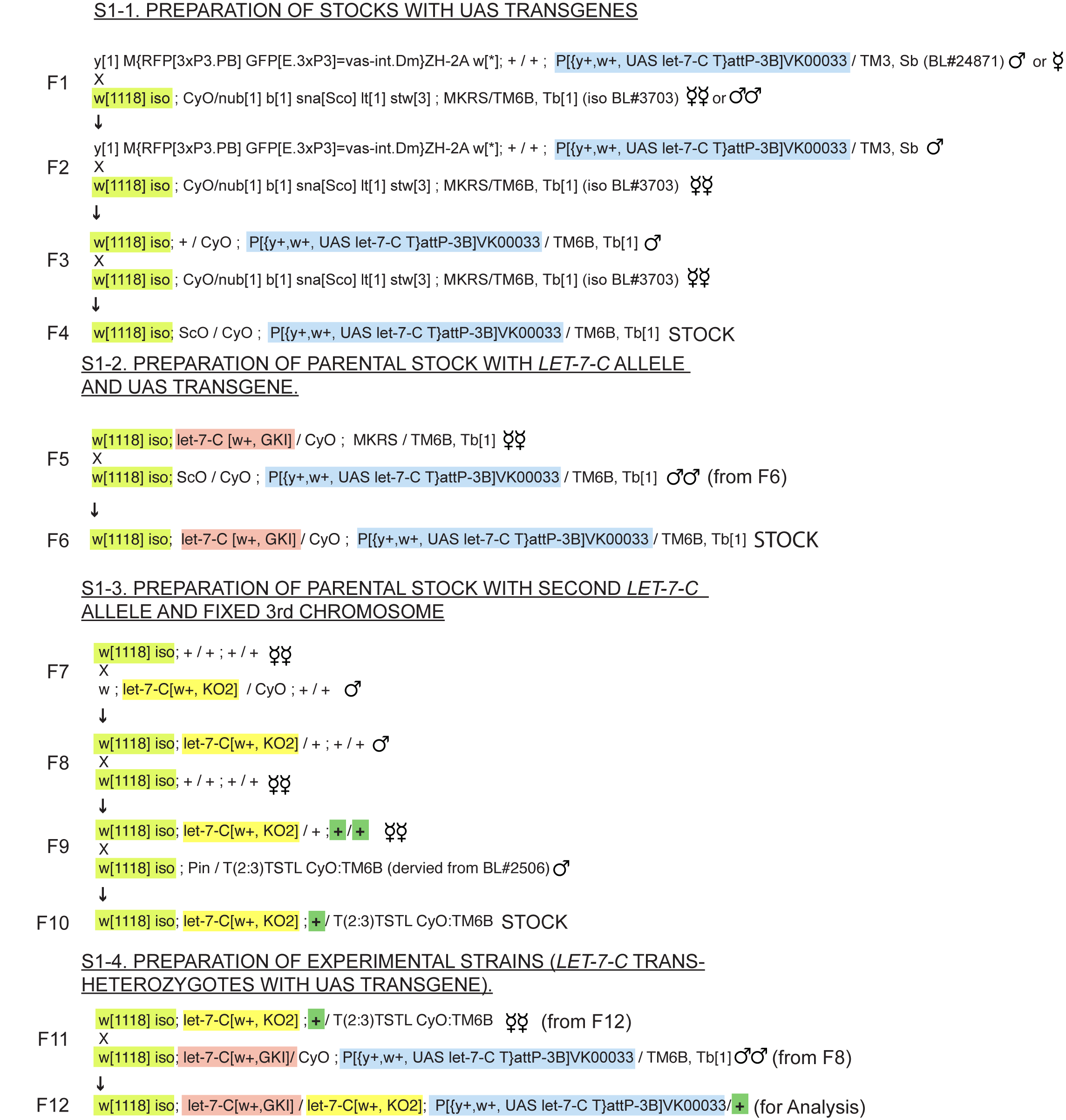

Supplement: Supplementary file 5 [file Image1.TIF]
