# Supplementary material for: Molecular Dissection of a Conserved Cluster of miRNAs Identifies Critical Structural Determinants That Mediate Differential Processing
Source: Front Cell Dev Biol. 2022 Jun 17;10:909212. doi: 10.3389/fcell.2022.909212 (PMC9247461; doi:10.3389/fcell.2022.909212)
Supplement: Supplementary file 6 [file Table2.DOCX]

**Supplementary Table 2.** Primers used in the study.

| **Name** | **Sequence** |
| --- | --- |
| Nhe1 miR-100 hairpin For | CTAGCacttgaaatggtgcatacttacatatggaccattaacagaaacccgtaaatccgaacttgtgctgttttatatctgttacaagaccggcattatgggagtctgtcaatgcaaacaactggtttttggcaacaaaatcaatgacaaaG |
| Nhe1 miR-100 hairpin Rev | CTAGCtttgtcattgattttgttgccaaaaaccagttgtttgcattgacagactcccataatgccggtcttgtaacaGatataaaacagcacaagttcggatttacgggtttctgttaatggtccatatgtaagtatgcaccatttcaagtG |
| Nhe1 let-7 hairpin For | CTAGCaaaccacctagcaaaaaggactacaccaaggacctttttctctctggcaaattgaggtagtaggttgtatagtagtaattacacatcatactatacaatgtgctagctttctttgcttgactacaagccgcatttgatG |
| Nhe1 let-7 hairpin Rev | CTAGCatcaaatgcggcttgtagtcaagcaaagaaagctagcacattgtatagtatgatgtgtaattactactatacaacctactacctcaatttgccagagagaaaaaggtccttggtgtagtcctttttgctaggtggtttG |
| Nhe1 miR-125 hairpin For | CTAGCaatattggcattggtgacatgtgcaaatgtttgtatggctgattccctgagaccctaacttgtgacttttaataccagtttcacaagttttgatctccggtattggacgcaaacttgctgatgttagtaaaaaataaggcaaG |
| Nhe1 miR-125 hairpin Rev | CTAGCttgccttattttttactaacatcagcaagtttgcgtccaataccggagatcaaaacttgtgaaactggtattaaaagtcacaagttagggtctcagggaatcagccatacaaacatttgcacatgtcaccaatgccaatattG |
| Nhe1 let-7 Hmir125LFor | CTAGCaaaccacctagcaaaaaggactacaccaaggacctttttctctctggcaaattgaggtagtaggttgtatagtacttttaataccagtttactatacaatgtgctagctttctttgcttgactacaagccgcatttgatG |
| Nhe1 let-7 Hmir125LRev | catcaaatgcggcttgtagtcaagcaaagaaagctagcacattgtatagtaaactggtattaaaagtactatacaacctactacctcaatttgccagagagaaaaaggtccttggtgtagtcctttttgctaggtggtttgctag |
| Nhe1let-7H miR100L For | CTAGCAaaccacctagcaaaaaggactacaccaaggacctttttctctctggcaaattgaggtagtaggttgtatagtactgttttatatctgttactatacaatgtgctagctttctttgcttgactacaagccgcatttgatG |
| Nhe1let-7H miR100L Rev | catcaaatgcggcttgtagtcaagcaaagaaagctagcacattgtatagtaacagatataaaacagtactatacaacctactacctcaatttgccagagagaaaaaggtccttggtgtagtcctttttgctaggtggtttgctag |
| Nhe1miR-125Hlet-7L For | CTAGCaatattggcattggtgacatgtgcaaatgtttgtatggctgattccctgagaccctaacttgtgagtaattacacatcatcacaagttttgatctccggtattggacgcaaacttgctgatgttagtaaaaaataaggcaaG |
| Nhe1miR-125Hlet-7L Rev | cttgccttattttttactaacatcagcaagtttgcgtccaataccggagatcaaaacttgtgatgatgtgtaattactcacaagttagggtctcagggaatcagccatacaaacatttgcacatgtcaccaatgccaatattgctag |
| Nhe1 miR125H miR100L For | CTAGCaatattggcattggtgacatgtgcaaatgtttgtatggctgattccctgagaccctaacttgtgactgttttatatctgttcacaagttttgatctccggtattggacgcaaacttgctgatgttagtaaaaaataaggcaaG |
| Nhe1 miR-125H miR100L Rev | cttgccttattttttactaacatcagcaagtttgcgtccaataccggagatcaaaacttgtgaacagatataaaacagtcacaagttagggtctcagggaatcagccatacaaacatttgcacatgtcaccaatgccaatattgctag |
| Nhe1 miR-100H miR-125L For | CTAGCacttgaaatggtgcatacttacatatggaccattaacagaaacccgtaaatccgaacttgtgcttttaataccagtttacaagaccggcattatgggagtctgtcaatgcaaacaactggtttttggcaacaaaatcaatgacaaaG |
| Nhe1 miR-100H miR-125L Rev | ctttgtcattgattttgttgccaaaaaccagttgtttgcattgacagactcccataatgccggtcttgtaaactggtattaaaagcacaagttcggatttacgggtttctgttaatggtccatatgtaagtatgcaccatttcaagtgctag |
| Nhe1 miR-100H let-7L For | CTAGCacttgaaatggtgcatacttacatatggaccattaacagaaacccgtaaatccgaacttgtggtaattacacatcatacaagaccggcattatgggagtctgtcaatgcaaacaactggtttttggcaacaaaatcaatgacaaG |
| Nhe1 miR-100H let-7L Rev | ctttgtcattgattttgttgccaaaaaccagttgtttgcattgacagactcccataatgccggtcttgtatgatgtgtaattaccacaagttcggatttacgggtttctgttaatggtccatatgtaagtatgcaccatttcaagtgctag |
| Nhe1 let-7H mir125B For | CTAGCaatattggcattggtgacatgtgcaaatgtttgtatggctgattgaggtagtaggttgtatagtagtaattacacatcatactatacaatgtgctagctttctggacgcaaacttgctgatgttagtaaaaaataaggcaaG |
| Nhe1 let-7H mir125B Rev | cttgccttattttttactaacatcagcaagtttgcgtccagaaagctagcacattgtatagtatgatgtgtaattactactatacaacctactacctcaatcagccatacaaacatttgcacatgtcaccaatgccaatattgctag |
| Nhe1 let-7H miR100BFor | CTAGCacttgaaatggtgcatacttacatatggaccattaacagatgaggtagtaggttgtatagtagtaattacacatcatactatacaatgtgctagctttcagtctgtcaatgcaaacaactggtttttggcaacaaaatcaatgacaaaG |
| Nhe1 let-7H miR100B Rev | ctttgtcattgattttgttgccaaaaaccagttgtttgcattgacagactgaaagctagcacattgtatagtatgatgtgtaattactactatacaacctactacctcatctgttaatggtccatatgtaagtatgcaccatttcaagtgctag |
| Nhe1 miR-125H let-7B For | CTAGCaaaccacctagcaaaaaggactacaccaaggacctttttctctctggcaaattccctgagaccctaacttgtgacttttaataccagtttcacaagttttgatctccggtattttgcttgactacaagccgcatttgatG |
| Nhe1 miR-125H let-7B Rev | CatcaaatgcggcttgtagtcaagcaaaataccggagatcaaaacttgtgaaactggtattaaaagtcacaagttagggtctcagggaatttgccagagagaaaaaggtccttggtgtagtcctttttgctaggtggtttGCTAG |
| Nhe1 miR-125H miR100B For | CTAGCacttgaaatggtgcatacttacatatggaccattaacagatccctgagaccctaacttgtgacttttaataccagtttcacaagttttgatctccggtatagtctgtcaatgcaaacaactggtttttggcaacaaaatcaatgacaaaG |
| Nhe1 miR-125H miR100B Rev | ctttgtcattgattttgttgccaaaaaccagttgtttgcattgacagactataccggagatcaaaacttgtgaaactggtattaaaagtcacaagttagggtctcagggatctgttaatggtccatatgtaagtatgcaccatttcaagtgctag |
| Nhe1 miR-100H miR-125B For | CTAGCaatattggcattggtgacatgtgcaaatgtttgtatggctggaaacccgtaaatccgaacttgtgctgttttatatctgttacaagaccggcattatgggtggacgcaaacttgctgatgttagtaaaaaataaggcaaG |
| Nhe1 miR-100H miR-125B Rev | cttgccttattttttactaacatcagcaagtttgcgtccacccataatgccggtcttgtaacagatataaaacagcacaagttcggatttacgggtttccagccatacaaacatttgcacatgtcaccaatgccaatattgctag |
| Nhe1 miR-100H let-7B For | CTAGCaaaccacctagcaaaaaggactacaccaaggacctttttctctctggcaaGAaacccgtaaatccgaacttgtgctgttttatatctgttacaagaccggcattatgggtttgcttgactacaagccgcatttgatG |
| Nhe1 miR-100H let-7B Rev | catcaaatgcggcttgtagtcaagcaaacccataatgccggtcttgtaacagatataaaacagcacaagttcggatttacgggtttcttgccagagagaaaaaggtccttggtgtagtcctttttgctaggtggtttgctag |
| del miR-100 avr2 for | gaccgccctaatgatttcttataCCTAGGtcgaaaaatcgacaaatggcggagtaagg |
| del miR-100 avr2 rev | ccttactccgccatttgtcgatttttcgaCCTAGGtataagaaatcattagggcggtc |
| del let-7 xba 1 for | gatatccagaagatcctttaaataccTCTAGAaaaagaatcccaatcgaactgcaccac |
| del let-7 xba1 rev | gtggtgcagttcgattgggattcttttTCTAGAggtatttaaaggatcttctggatatc |
| del miR125 spe1 for | gtatgtaaatgcaaccgggcatatgtaACTAGTaaacgaattccctttcaaggcac |
| del mir125 spe 1 rev | gtgccttgaaagggaattcgtttACTAGTtacatatgcccggttgcatttacatac |
| Xho1 let-7 cDNA For | CCGCTCGAGTTGCACACGGCGGTTCGCGAATCGCG |
| Kpn 1 let-7 cDNA Rev with Xba deleted | CGGGGTACCacttagaagtagcttgtagtttaaataca |
| litmus Rev-2 Xba I  (2162) | gccttgactagagggtaccagagctcacctagg |
| T7 +Pri-miR-100 For  (2175) | CGTTAATACGACTCACTATAGGacttgaaatggtgcatacttacatatgg |
| miR-100 BamHI Rev  (2186) | GATCGGATCCtttgtcattgattttgttgccaaaaaccag |
| ds of miR-125 Rev (2164) | ggagaacttctctgaattgccgatacttgtg |
